# Vulnerability of older adults to heatwaves: the Barcelona case

**DOI:** 10.3389/fpubh.2026.1808985

**Published:** 2026-08-14

**Authors:** Blanca Arellano, Josep Roca, Carina Serra, Dolors Martínez

**Affiliations:** 1 Center for Land Policy and Valuation, Department of Architectural Technology, Universitat Politècnica de Catalunya, Barcelona, Spain; 2 Center for Land Policy and Valuation, Department of Physics, Universitat Politècnica de Catalunya, Barcelona, Spain

**Keywords:** older adults, global warming, health heat waves, heat-related mortality, minimum temperatures, nighttime heat waves

## Abstract

Heatwaves are becoming more frequent, intense, and persistent as a result of global warming, posing a growing public health challenge in Mediterranean cities. In Barcelona, this process is characterized by a pronounced increase in nighttime temperatures, amplified by the urban heat island effect and by a rapidly aging population, which together increase vulnerability to heat-related health impacts. This study aims to assess the effects of high temperatures on mortality in Barcelona, with a particular focus on older adults, and to determine the relative importance of nighttime vs. daytime heat. To this end, the association between temperature and daily mortality was analyzed using time-series epidemiological methods. Health heat waves (HHW) were defined based on epidemiologically derived temperature–mortality relationships and persistence criteria, distinguishing between daytime (DHHW) and nighttime (NHHW) events. Generalized additive models were applied at the city scale to identify health-relevant temperature thresholds and HHW events, while generalized linear models were used at the provincial scale to evaluate temperature-related mortality risks among the older adults. Results show that nighttime temperatures play a more decisive role than daytime temperatures in shaping heat-related mortality in Barcelona. Nighttime health heat waves are more frequent, longer-lasting, and associated with substantially higher mortality than daytime events. Between 2007 and 2018, 721 deaths were attributable to NHHW, compared to 571 deaths attributable to DHHW. Provincial analyses further confirm that both maximum and minimum temperatures significantly increase mortality among older adults, although nighttime effects are likely underestimated due to spatial aggregation and the smoothing of extreme temperatures in reanalysis data. The main contribution of this study is to demonstrate that, in Barcelona, high nighttime temperatures constitute the most harmful thermal factor for human health, particularly among older adults, challenging heat–health frameworks that focus primarily on daytime extremes. These findings highlight the need to revise current heat–health warning systems by explicitly incorporating nighttime temperatures and persistence criteria, in order to improve prevention strategies in aging Mediterranean cities.

## 1 Introduction

Althoughx global warming (GW) is a well-known fact (1), its quantification is a subject of debate, both globally and regionally. In particular, there are doubts about whether the 1.5°C threshold set at the 2015 Paris summit has already been reached. For Copernicus, 2024 was a year of unprecedented global temperatures. It also became the first year with an average temperature clearly 1.5°C above pre-industrial levels (2). The global surface temperature for 2024 was 1.46°C−1.62°C above the 1,850–1,900 average (3). However, as the WMO has indicated, exceeding the 1.5°C threshold in a single year does not constitute a breach of the Paris Agreement limit; this would require exceeding +1.5°C over a longer period (4). Currently, the quantification of GW is estimated to be between 1.35°C (5) and 1.42°C (6) with respect to the pre-industrial era.

The Mediterranean area, and Spain in particular, is experiencing a temperature increase well above the global average. Over the past few decades, a substantial body of research has indicated that the rate of temperature increase in the region is significantly faster than the global average (7–10). This has led to a marked increase in heat waves, which have a significant impact on human health. The warming process has been pronounced in mainland Spain and the Balearic Islands, which represent an actual hotspot of global warming. Spain has greatly exceeded the 1.5°C threshold compared to the pre-industrial era. The increase in temperatures between 1971 and 2024^1^ in mainland Spain and the Balearic Islands was 1.96°C (11, 12), which is well above the world average (1.29°C) and even above the average of the Mediterranean area (1.83°C), if the historical estimates of CMIP6 are taken as a reference^2^ (https://atlas.climate.copernicus.eu/atlas). The temperature increase in Spain is expected to continue rising in the coming decades, likely replacing the traditional Mediterranean climate (Csa in the Köppen classification) with a warmer and, generally, drier climate (13). This climate change and global warming are increasingly associated with reductions in thermal comfort and the health of urban populations.

GW increases the likelihood of extreme temperatures, as well as heat waves (HW). Empirical data suggest that both the frequency and intensity of heat waves have increased in Europe (14), with Spain being one of the most severely affected countries (15). Heat waves constitute one of the most important climate-related hazards affecting human health. Numerous epidemiological studies have demonstrated a robust association between extreme heat events and increases in all-cause mortality, as well as cardiovascular, respiratory, and renal morbidity (16–18). Heat-related mortality often occurs rapidly during HW episodes and is frequently underestimated, as deaths are commonly attributed to underlying chronic conditions rather than to heat exposure itself (19, 20). The health impacts of heat waves are not evenly distributed across the population. Older adults constitute the most vulnerable group to extreme heat due to age-related physiological changes, the higher prevalence of chronic diseases, and the frequent use of medications that interfere with thermoregulation (21–23). Numerous studies conducted in Europe and Spain have consistently shown that heat-related excess mortality increases sharply with age, particularly among individuals aged 65 years and older (24, 25). Within this age group, women have been identified as a particularly vulnerable subgroup, due to a combination of biological, demographic, and social factors, including longer life expectancy, a higher likelihood of widowhood and living alone, and a greater exposure to economic deprivation in old age (26–29).

Despite the relevance of heat waves for public health, there is no universally accepted definition of HW. In Spain, operational definitions are generally based on meteorological thresholds, most commonly using maximum daily temperature (TX) percentiles over a given reference period, combined with a minimum duration criterion (30–32). These definitions are widely used in heat-health warning systems and form the basis of national and regional prevention plans. The most widely used definition of a HW in Spain is that of AEMET (33): “A heat wave is considered to be an episode of at least three consecutive days, in which the station considered record maximums above the 95th percentile of their series of daily maximum temperatures for the months of July and August of the period 1971–2000.”

The predominance of maximum temperature in HW definitions reflects its strong association with daytime heat stress and its historical availability in meteorological records. Consequently, most epidemiological studies and warning systems have focused primarily on TX as the main explanatory variable of heat-related health outcomes (34, 35). However, this approach may overlook important aspects of thermal exposure, particularly during nighttime periods. In recent years, increasing attention has been paid to the role of minimum temperature (TN) and nighttime heat in shaping heat-related health risks. Several studies have shown that elevated nighttime temperatures can be equal or even more harmful than daytime extremes, as they impede physiological recovery and increase cumulative heat stress (36–38). In Spain, research has highlighted the significant contribution of high TN to excess mortality, especially among older adults and during prolonged heat events (39, 40).

This growing body of evidence has led to the introduction of the concept of nighttime heat waves (NHW), which refer to periods of persistently elevated minimum temperatures. The most commonly used conventional definition of a nighttime heat wave in Spain consists of adapting the AEMET definition (33) to the TN: “A heat wave is considered to be an episode of at least three consecutive nights above the 95th percentile of the daily minimum temperatures of the months of July and August of the period 1971–2000” (41). Nighttime heat waves differ from conventional daytime heat waves not only in their thermal characteristics but also in their physiological and epidemiological implications, particularly for vulnerable populations (42). Nevertheless, NHW remain underrepresented in heat-health warning systems and are rarely considered explicitly in operational definitions.

In parallel with advances in heat-health research, several authors have proposed moving beyond purely meteorological definitions of heat waves toward a health-based framework, introducing the concept of health heat waves (HHW). This approach defines heat waves according to their observed impact on population health, typically through statistically significant increases in mortality or morbidity above expected baseline levels, rather than solely by climatological thresholds (43, 44). In Spain, this epidemiological perspective has been explicitly incorporated into the development of heat-health surveillance and warning systems, where temperature thresholds are derived from mortality-temperature relationships and tailored to regional vulnerability patterns. More recently, research conducted by ISGlobal and collaborating institutions has reinforced the relevance of health-based heat wave definitions, highlighting their utility for identifying high-risk periods, particularly among older adults and in urban environments affected by nocturnal heat exposure (38, 39, 45).

Urban environments further exacerbate heat exposure through the urban heat island (UHI) effect, whereby built-up areas experience higher temperatures than their surrounding rural regions, especially during nighttime hours (41, 46–48). The UHI effect is particularly relevant in compact Mediterranean cities, where high building density, limited green space, and anthropogenic heat emissions contribute to sustained nocturnal warmth (49, 50). As a result, urban populations -and older adults in particular- are exposed to higher minimum temperatures and prolonged heat stress, increasing the risk of adverse health outcomes.

Within the aforementioned theoretical framework, this article aims to determine the effects of high temperatures, especially nighttime temperatures, on the health of older adults in the city of Barcelona. To this end, its main theoretical challenge is to establish an operational definition of a health heat wave (HHW) that goes beyond a purely meteorological perspective. Furthermore, it seeks to differentiate, within this perspective, between daytime (DHHW) and nighttime (NHHW) health heat waves, proposing the hypothesis that, in Barcelona, NHHW have a greater impact on health than DHHW, particularly on the health of older adults.

## 2 Data and methods

### 2.1 Databases

The databases used in this work are the following:

- Regarding the demographic analysis of Barcelona and its province (sex and age groups), information from IDESCAT, the statistical office of Catalonia, has been used. This information is both retrospective (1975–2025), https://www.idescat.cat/pub/?id=ep&n=7741&t=201802&geo=prov:08&lang=en) and prospective (2024-2044, https://www.idescat.cat/novetats/?id=5285&lang=en).
- Regarding climate information, for the city of Barcelona the Raval meteorological station has been used (41), relating to maximum (TX), minimum (TN) and average (TM) temperatures, from January 1, 1971 to December 31, 2025, provided by the Servei Meteorològic de Catalunya (SMC, https://www.meteo.cat/observacions/xema/dades). Although there are three other official weather stations in Barcelona, the Raval station is the most representative of the city's climate, and as such is the one officially used by the SMC in estimating the city's temperatures.
- For meteorological information for the province of Barcelona, the TX, TN and TM data provided by ERA5 have been used. Since there is no official daily TX, TN and TM statistics from the Agencia Estatal de Meteorología (AEMET) or the SMC of the province of Barcelona, it has been necessary to resort to the reanalysis of ERA5 (https://www.ecmwf.int/en/forecasts/datasets/browse-reanalysis-datasets). The climate data obtained covers the period from January 1, 2015 to December 31, 2025.
- For the construction of the mortality models and the obtaining of the daytime and nighttime health heat waves (DHHW & NHHW) of the city of Barcelona, a daily database has been used, between January 1, 2007 and December 31, 2018, provided by the Barcelona Health Agency.
- To obtain the mortality models for the older adults, the MoMo database from the Carlos III Health Institute (part of the Spanish Ministry of Health) was used. This database was obtained for all calendar days from January 1, 2015, to December 31, 2025, disaggregated for the different age groups, for the province of Barcelona (https://momo.isciii.es/panel_momo/).

The attached Table 1 summarizes the databases used, including their source, spatial resolution, and temporal coverage.

**Table 1:** Databases.

Database	Spatial resolution	Temporal coverage	Source
Demographic information	Barcelona & Province	1975–2025	IDESCAT
Age pyramid	Barcelona	2021	INE
Climate Information	Barcelona	1971–2025	SMC
Climate Information	Province	2015–2025	ERA5
Daily mortality	Barcelona	2007–2018	ASB
Daily mortality	Province	2015–2025	ISCIII

### 2.2 Study area

The study area is the city of Barcelona (41° 3′ 19.68” N, 2° 9′ 32.36” E). Barcelona is the capital of the Autonomous Community of Catalonia and the second most populous city in Spain. It has an area of 101.35 km^2^ and a population (2025) of 1,731,649 inhabitants. Located on the Mediterranean coast, it has an average altitude of 13 m above sea level. It occupies the central place of the so-called “Pla de Barcelona,” and the heart of its Metropolitan Area.

**Figure 1:**
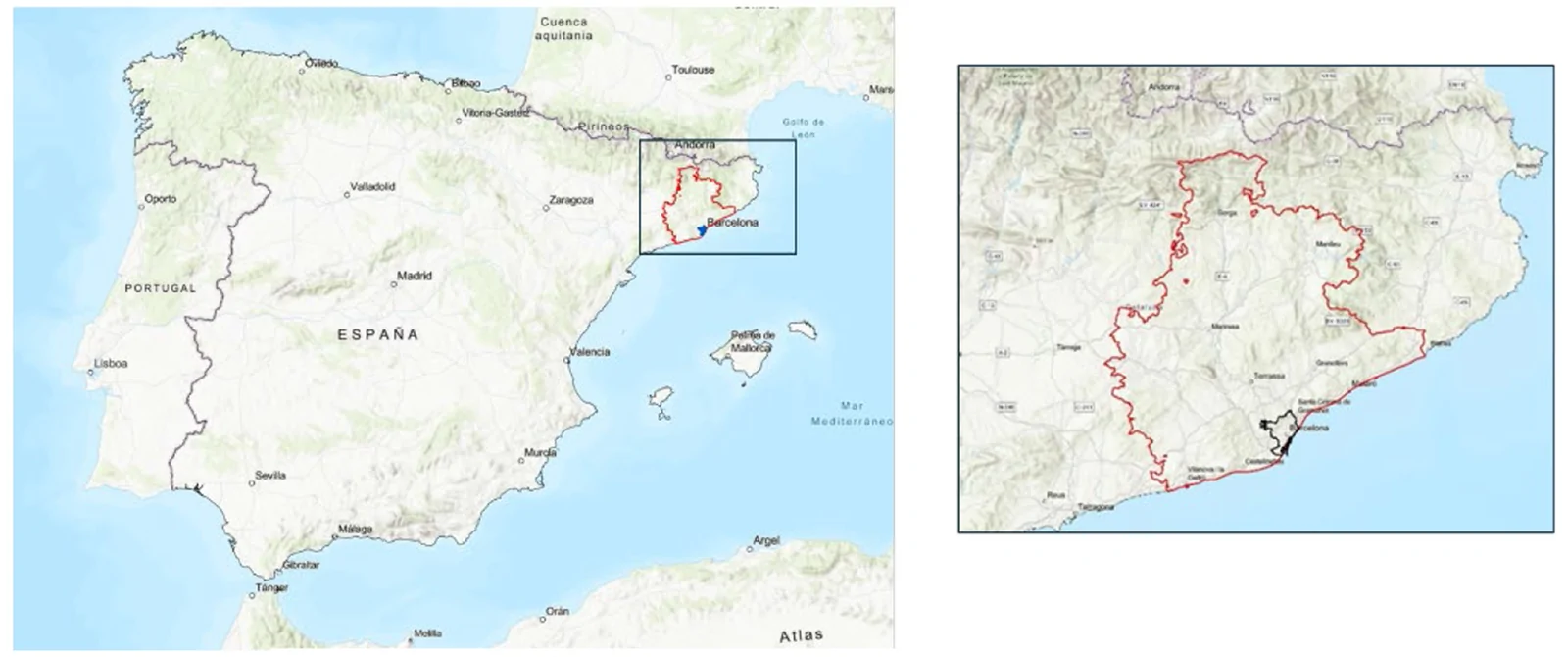
Barcelona: municipality and province.

The province of Barcelona, with an area of 7,726 km^2^ and a population of 5,959,941 inhabitants (2025), comprises 311 municipalities and has a very rugged topography (the average altitude of the province of Barcelona is 469 meters, with a variation between 0 and 2,536 meters above sea level), which significantly influences the climate, ranging from the sea (where the capital is located) to the Pyrenees. This includes diverse geographical areas such as the Coastal Depression, the Coastal Range, the Pre-Coastal Depression, the Pre-Coastal Range, the Central Depression, the Pre-Pyrenees, and the Pyrenees. Figure 1 presents the study area.

### 2.3 Methodology

#### 2.3.1 Estimation of the population vulnerable in Barcelona to the warming process

Since the objective of the study is to estimate the vulnerability of the older adults to the warming process, the evolution of the population ≥ 64 years in the course of the last decades (1975–2025) will be quantified, as well as establishing the degree of aging of Barcelona in the near future (2050). The feminization of old age, especially among older age groups (>= 75–80 years), is a focus of attention, given their greater health, economic and social vulnerability. Official statistical sources (IDESCAT) are used for this purpose.

Given that Barcelona's population is experiencing high growth due to immigration, the progressive aging of the city's population is masked by the influx of immigrants. Therefore, the percentage of older adults people relative to the total population is not a suitable indicator for measuring the true degree of aging in Barcelona, and consequently, the extent of its vulnerable population. This paper emphasizes the importance of estimating real population contingents, according to age groups, instead of using ratios.

The estimate of the older adults population in the horizon year of this study (2050) is made according to official criteria. As this research is not specifically aimed at establishing a prospective demographic analysis, and given the great uncertainty of the migration process,^3^ the most recent projection made by IDESCAT for the period 2024–2044, extrapolated (OLS) to 2050, has been used. However, in section 4.1, regarding the discussion of the results, the probable underestimation of the degree of aging of the city, obtained from the current official projection, is argued.

#### 2.3.2 Estimation of the Barcelona warming process

Using climate data from the Raval station as the most representative of Barcelona (41), the degree of warming experienced by the city between 1971 and 2025 is determined, as well as a prospective estimate to 2050. Since one of the main objectives of the research is to test the hypothesis of greater nighttime warming, given the geographical location of Barcelona, as well as the urban heat island (UHI) effect (more pronounced at night than during the day), the evolution of TN is differentiated from TX.

The determination of extreme heat events in Barcelona is a key focus of this work. Given the lack of academic consensus on how to define HW, the official definition (33) from AEMET (the Spanish State Meteorological Agency) is adopted, which states that a heat wave occurs when the temperature exceeds the 95th percentile (P95) of the reference period (1971–2000) for three or more consecutive days. This definition is unique in that it considers not only daytime heat waves (DHW, when TX > P95) but also nighttime heat waves (NHW, when TN > P95) (41).

The analysis of warming trends is performed using ordinary least squares regression (OLS), and using the more robust non-parametric Kendall-Theil-Sen regression (KTS).^4^ The Mann-Kendall test (MK) and Kendall's tau (τ) is used to determine the degree of statistical confidence in the correlations between temperatures and the passage of time. In this study, temporal trends were obtained (by OLS and KTS regression models) using as dependent variables the temperature (TG, TX, TN, DHW and NHW) and the year as the independent variable. The Sen's slope resulting from the KTS regressions and the *p*-value of the MK test allow an evaluation of the positive or negative trend and the statistical significance of the developed models. The Sen's slope resulting from the KTS models was used to estimate the trend between 1971 and 2025 and the projections up to 2050 (51). So, the estimation of the future climate (2050) is therefore not carried out using conventional climate modeling (CMIP6), but rather by extrapolating trends (Sen's slope) observed from 1971 to the present, if the confidence level (*p*-value) exceeds 95%. Following the methodology established by the IPCC (1), if the *p*-value < = 0.05, it is considered “extremely likely.”

Statistical projection using Kendall-Theil-Sen (KTS) regression, supported by the Mann-Kendall test, is used to ensure the robustness and statistical significance of the observed trends. This approach is intended to estimate the continuation of empirically observed warming patterns over the period 1971–2025, rather than to provide a full climate projection based on physically driven climate model scenarios. The use of statistical methods is particularly suitable in this case due to the availability of a long, high-quality local dataset and the need to capture site-specific processes such as the Urban Heat Island (UHI), which may not be fully resolved in regional or global climate models.

However, this approach entails important limitations that must be acknowledged. In particular, it assumes temporal persistence and linearity of past trends and does not explicitly account for future changes in greenhouse gas emissions, large-scale circulation patterns, or potential non-linear responses of the climate system. Therefore, the projections presented here should be interpreted as indicative, trend-based scenarios that complement, rather than replace, climate model-based projections. Future research should aim to integrate both approaches in order to provide a more comprehensive assessment of climate change impacts at the urban scale.

In summary, given that the present research has as the hypothesis that high nighttime temperatures represent the greatest risk to the health of citizens in Barcelona, and especially the health of the older adults, the main focus is on comparing daytime and nighttime warming trends, with special emphasis on TN and NHW, in relation to TX and DHW.

#### 2.3.3 To assess the impact of high temperatures on mortality and identify healthcare heat waves (HHW) in the city of Barcelona

To assess the impact of high temperatures on mortality in Barcelona and to identify health heat waves (HHW), a multi-step analytical approach was adopted, combining exploratory statistical modeling and epidemiological time-series methods. Particular emphasis was placed on contrasting the effects of maximum temperature (TX) and minimum temperature (TN), in order to test the hypothesis that nighttime temperatures exert a stronger influence on mortality than daytime temperatures in the urban context of Barcelona.

As a first step, the general relationship between temperature and daily mortality was explored over the entire study period using polynomial regression models. Curvilinear models were fitted to identify temperature ranges associated with minimum mortality and to detect the inversion point beyond which mortality increases with temperature. This exploratory analysis allowed the identification of TX and TN thresholds at which the temperature–mortality relationship changes sign trend, providing an initial indication of differential impacts between both temperatures.

Subsequently, to isolate the effect of high temperatures on mortality during the warm season, the analysis focused on summer conditions, defined by days with TX ≥ 25°C (“summer days”) and nights with TN ≥ 20°C (“tropical nights”). Quadratic and cubic models were used to characterize the seasonal pattern of mortality and to estimate expected baseline mortality for each calendar day. Excess summer mortality was then quantified as the deviation of observed daily deaths from this expected seasonal baseline.

Formally, expected daily mortality was estimated as:


$$\begin{array}{l} \hat{Y_{t}}=f\left(d_{t}\right) \end{array}$$

Where $\hat{Y_{t}}$ is the expected number of deaths on day *t*, and *f*(*d*_*t*_) is a cubic function of the calendar day controlling for seasonal variability. Excess mortality was computed as the residual:


$$\begin{array}{l} \varepsilon_{t}=Y_{t}-\hat{Y_{t}} \end{array}$$

where *Y*_*t*_ represents observed daily mortality.

To assess the specific contribution of temperature to summer^5^ excess mortality, ordinary least squares (OLS) regression models were fitted between the residuals ε_*t*_ and TX and TN, respectively. This approach allowed a direct comparison of the explanatory power and regression coefficients associated with daytime and nighttime temperatures.

In a second stage, temperature–mortality associations were modeled using generalized additive models (GAMs) with a Poisson distribution and a logarithmic link function, which are well suited for count data and non-linear exposure–response relationships (35). The general form of the model is given by:


$$\begin{array}{l} ln\left(E\left(Y_{t}\right)\right)=\alpha +s\left(T_{t}\right)+s\left(time_{t}\right)+DOW_{t} \end{array}$$

where *E*(*Y*_*t*_) is the expected number of deaths on day *t*, α is the intercept, *s(T*_*t*_*)* is a smooth function of temperature (TX or TN), *s*(*time*_*t*_) is a smooth function controlling for long-term trends and seasonality, and *DOW*_*t*_ is a categorical variable accounting for day-of-week effects.

From these models, temperature-specific daily relative risks (*RR*_1*d*_) were derived as:


$$\begin{array}{l} RR_{1d}\left(T\right)=\exp\left(\beta \left(T\right)\right) \end{array}$$

where β(*T*) represents the estimated temperature effect. Thresholds corresponding to predefined relative risk levels (RR = 1.05, 1.10, and 1.15) were identified for both TX and TN. All relative risks (RR) were computed relative to a reference temperature corresponding to the 50th percentile (P50) of the observed temperature distribution. An RR of 1.1, for example, indicates a 10% increase in mortality risk relative to the reference temperature.

To define health heat waves (HHW), it was necessary to move beyond isolated daily risk and incorporate the concept of persistence, which is central to public-health relevance. A heat-health wave was therefore defined as a period of at least *k* consecutive days (or nights) during which temperature exceeds a given threshold. The primary definition adopted in this study corresponds to *k* ≥ *3d*, consistent with the operational logic of heat–health warning systems.

Within this episodic framework, cumulative relative risk over three consecutive days *RR*_3*d*_ was defined as:


$$\begin{array}{l} RR_{3d}=\overset{3}{\underset{i=1}{\prod }}RR_{1d,i} \end{array}$$

Thresholds were recalibrated to identify temperature values associated with an equivalent episodic risk of *RR*_3*d*_ = 1.10. This procedure yields lower and more operationally meaningful thresholds than those obtained from daily risk alone, allowing direct comparison with official warning criteria.

Two types of health heat waves were identified: daytime health heat waves (DHHW), based on TX thresholds, and nighttime health heat waves (NHHW), based on TN thresholds. For each type, the number of events, their duration, and the associated excess mortality were quantified. This methodological framework provides a robust basis to test the central hypothesis of the study, namely that nighttime temperatures are more strongly associated with heat-related mortality than daytime temperatures in the city of Barcelona.

#### 2.3.4 Estimate the effect of high temperatures on the health of the older adults in the province of Barcelona

The analysis of the impact of high temperatures on older adults mortality conducted for the city of Barcelona is limited by the lack of daily mortality data disaggregated by age and sex at the municipal level. The mortality database used for the city does not provide this level of detail. Consequently, to assess the specific effects of high temperatures on the health of older adults, the analysis was extended to the *provincial scale*.

Daily mortality data disaggregated by age group were obtained for the province of Barcelona from the Spanish Daily Mortality Monitoring System (MoMo) (https://momo.isciii.es/panel_momo/). The study period spans from January 1, 2015 to December 31, 2025. As a precautionary measure, the year 2020 was excluded from the analysis due to the exceptional impact of the COVID-19 pandemic on mortality patterns. The analysis was restricted to the summer season (June to September), when heat-related mortality effects are most pronounced.

Daily maximum (TX) and minimum (TN) temperatures were obtained from the *ERA5 reanalysis dataset*. Reanalysis data were used because official meteorological statistics from AEMET and the Meteorological Service of Catalonia (SMC) do not provide consistent daily temperature series at the provincial scale. ERA5 provides spatially and temporally homogeneous temperature fields, allowing the derivation of representative daily TX and TN values for the province of Barcelona. However, reanalysis products tend to smooth extreme temperature events and do not fully capture local-scale phenomena such as the urban heat island (UHI) effect, particularly during nighttime hours. This limitation is especially relevant in a geographically heterogeneous province such as Barcelona, where large variations in altitude, distance to the sea, and urbanization may differentially affect TX and TN. These issues are further addressed in the Discussion section. The provincial analysis estimates incremental temperature–mortality associations under heterogeneous climatic conditions and does not define health thresholds, which were addressed at the city scale.

To quantify the association between temperature and mortality, generalized linear models (GLM) with a Poisson distribution and a logarithmic link function were employed, an approach widely used in epidemiological time-series analyses of environmental exposures (17, 31, 52). Separate models were estimated for maximum temperature (TX) and minimum temperature (TN) in order to explicitly contrast their respective impacts on mortality and to test the central hypothesis of this study regarding the greater health relevance of nighttime temperatures.

In these models, the dependent variable was daily mortality among individuals aged 65 years and older. For comparative purposes, analogous models were also estimated for mortality in the total population. Temperature (TX or TN) was included as the main explanatory variable. To account for temporal confounding, models were adjusted for long-term trends using a continuous time variable, and for short-term variability associated with weekly cycles by including a categorical day-of-week (DOW) variable.

Daily mortality was assumed to follow a Poisson distribution:


$$\begin{array}{l} Y_{t}\sim \text{Poisson}\left(\mu_{t}\right) \end{array}$$

where *Y*_*t*_ is the observed number of deaths on day *t* and μ_*t*_ = E(*Y*_*t*_) is the expected number of deaths on day *t*.

The systematic component of the model was specified using a log-link function as follows:


$$\begin{array}{l} \log\left(\mu_{t}\right)=\alpha +\beta T_{t}+\gamma time_{t}+DOW_{t} \end{array}$$

where α is the intercept, *T*_*t*_ represents the daily temperature (TX or TN), β is the temperature-related regression coefficient, *time*_*t*_ is a continuous variable accounting for long-term temporal trends, and *DOWt* is a categorical variable controlling for day-of-week effects.

Separate models were estimated for maximum temperature (TX) and minimum temperature (TN) in order to explicitly contrast the effects of daytime and nighttime temperatures on mortality. From each model, the relative risk (RR) associated with a 1 °C increase in temperature^6^ was derived as:


$$\begin{array}{l} RR=\exp\left(\beta \right) \end{array}$$

For comparative purposes, analogous models were also estimated for mortality in the total population, allowing the assessment of differential temperature sensitivity between older adults and the population as a whole.

All models were implemented using the Python programming language, employing standard statistical libraries for generalized linear modeling. Model fit and statistical significance were assessed using conventional criteria, with a significance level of 0.05.

This provincial-scale modeling framework allows the estimation of temperature-related mortality risks among older adults in the absence of age-disaggregated city-level data and provides a consistent basis to test the central hypothesis of this study regarding the relative importance of minimum vs. maximum temperatures. The use of reanalysis data and the spatial aggregation inherent to the provincial scale may lead to an underestimation of extreme temperature exposure—particularly nighttime temperatures—due to the smoothing of local extremes and the partial omission of urban heat island effects. These limitations are considered in the interpretation of the results and further discussed in Section 4.4.

### 2.4 Methodological framework

The methodological framework adopted in this study follows an integrated and sequential approach that combines demographic analysis, climate characterization, epidemiological modeling, and health-risk assessment. This framework is designed to ensure coherence between data sources, analytical methods, and research objectives, and to explicitly address the vulnerability of older adults to heat exposure in an urban context.

The first analytical block focuses on the demographic evolution of the city of Barcelona, with particular attention to the aging process of its population. This block characterizes the current age structure and the temporal evolution of the population aged 65 years and over, and places special emphasis on future demographic projections up to 2050. The progressive increase in the proportion of older adults—especially among the oldest age groups—constitutes a key structural factor that amplifies heat-related health risks and provides the demographic basis for the subsequent analyses. Understanding this process is essential to contextualize the public health relevance of heat exposure in Barcelona and to frame the study within a long-term vulnerability perspective.

The second analytical block addresses long-term climate dynamics and thermal exposure. This stage characterizes trends in mean, maximum, and minimum temperatures and examines the evolution of extreme heat events over recent decades. Particular attention is paid to the differentiation between daytime and nighttime thermal extremes, given their distinct physical drivers and physiological implications. This block provides the climatic context necessary to interpret heat-related health outcomes and to identify relevant exposure patterns.

The third analytical block examines the relationship between temperature and mortality using time-series epidemiological methods. At the city scale, this analysis focuses on identifying temperature thresholds associated with increased mortality and on defining health heat waves (HHW) based on epidemiological criteria rather than purely meteorological definitions. This approach explicitly distinguishes between daytime health heat waves (DHHW) and nighttime health heat waves (NHHW), allowing a direct assessment of the relative contribution of maximum and minimum temperatures to heat-related mortality.

The fourth analytical block extends the analysis to the provincial scale in order to assess the specific vulnerability of older adults. Due to the lack of age-disaggregated daily mortality data at the municipal level, generalized linear models are applied to provincial mortality data by age group. This multiscale approach enables the quantification of the effects of maximum and minimum temperatures on older adults mortality during the summer season and provides a complementary perspective to the city-level analysis.

Across all analytical stages, the framework emphasizes the role of persistence, cumulative exposure, and spatial heterogeneity as key determinants of health risk. The use of reanalysis temperature data allows for spatially consistent assessments over extended periods, while acknowledging limitations related to the smoothing of extreme events and the underrepresentation of urban heat island effects, particularly during nighttime hours. These limitations are explicitly considered in the interpretation of the results.

**Figure 2:**
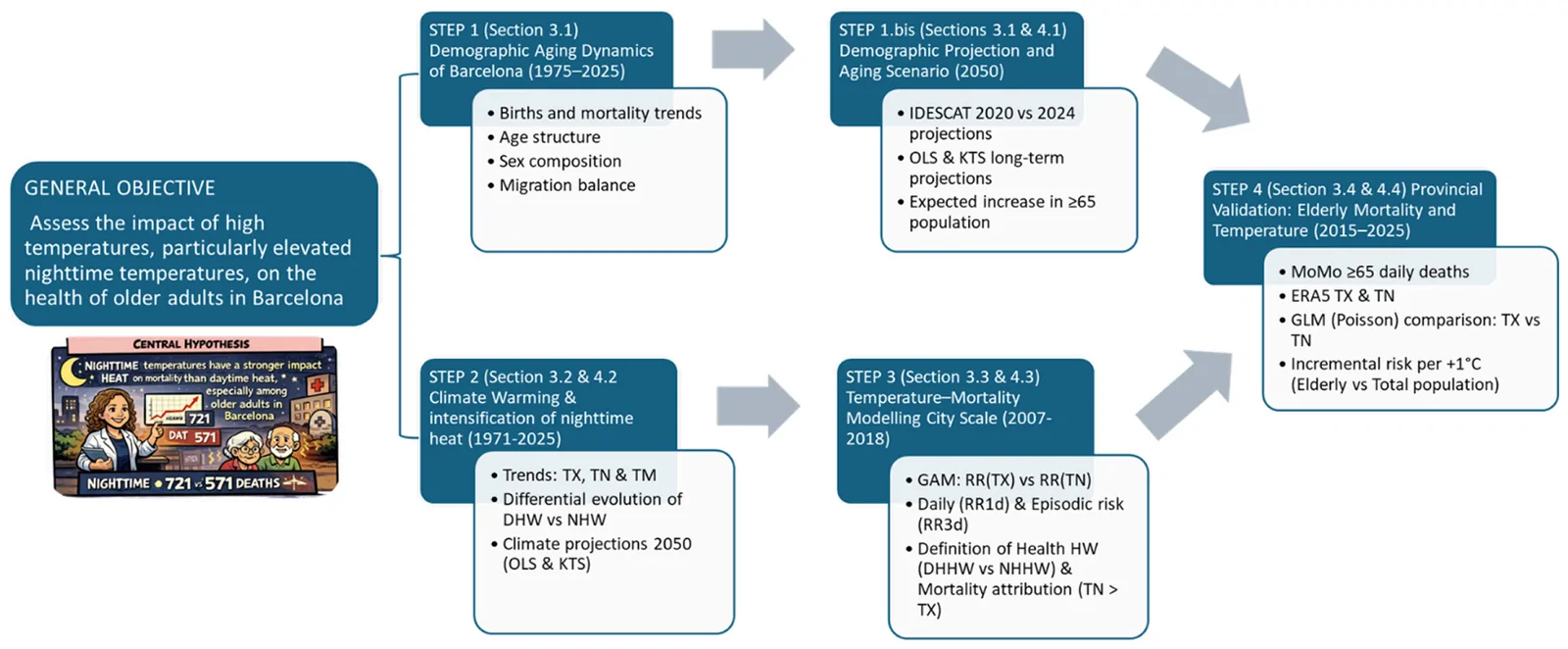
Methodological framework.

Overall, this methodological framework provides a coherent basis for integrating demographic change, climate dynamics, thermal exposure, and health outcomes. It allows for a systematic evaluation of the central hypothesis of this study: that nighttime temperatures and nighttime heat waves play a more decisive role than daytime extremes in shaping heat-related mortality in the urban context of Barcelona, particularly among older adults. Figure 2 summarizes the methodological framework of the research.

## 3 Results

### 3.1 Population aging

**Figure 3:**
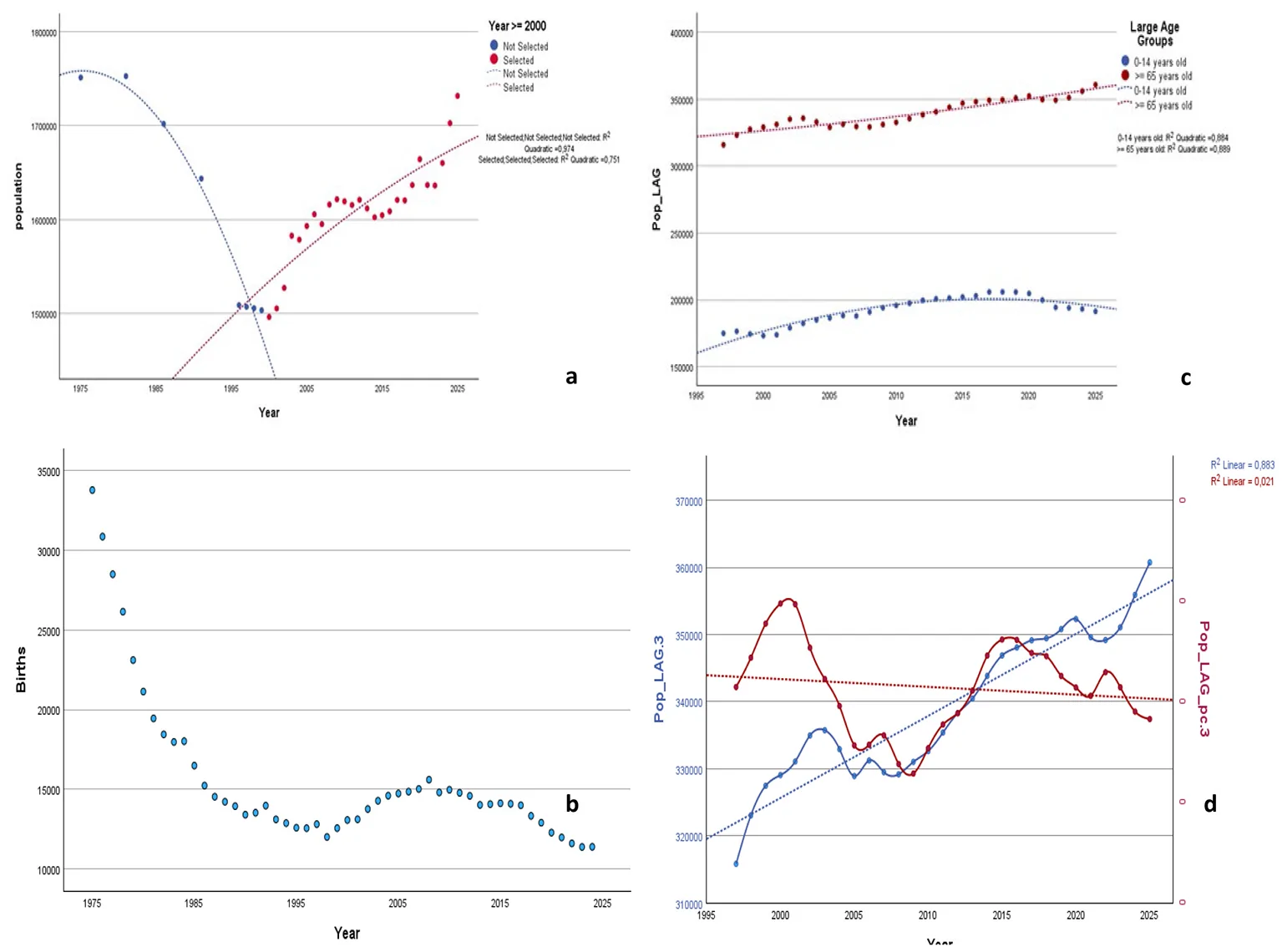
Demographic trends. **(a)** Population of Barcelona (1975–2025); **(b)** Number of births (1975–2024); **(c)** Older adults (≥65 years) vs. child (≤14 years); **(d)** Population and % of older adults. LAG means large age groups. LAG3 refers to the age group (≥65 years).

The population of the city of Barcelona has not stopped growing since the historic low of the year 2000 (1,496,266 inhabitants), when for the first time it fell below the threshold of one and a half million inhabitants. From then on, the declining population trend, resulting from the suburban migration process that had been occurring continuously since 1980, was reversed. Figure 3a shows the break in the demographic process that occurred around the year 2000. Table 2 shows the process of population decentralization that occurred in Barcelona between 1981 and 2001, a process that represented an unprecedented metropolitan expansion (53).

**Table 2:** Population of Barcelona and its hinterland (1981–2001).

Variable	1981	1986	1991	1996	2001
Pop. of Barcelona	1,752,627	1,701,812	1,643,542	1,508,805	1,505,325
Pop. Hinterland	1,402,360	1,470,452	1,563,450	1,662,659	1,636,242
N. municipalities (Hinterland)	37	55	66	86	89

From 1998–2000, with Spain's entry into the Eurozone and the resulting economic expansion, the massive influx of immigrants reversed the municipality's demographic decline, initiating a process of population recovery, until reaching the current demographic peak of the previous period (1975). In this sense, Barcelona follows the path laid out in major world cities, where a process of population recentralization has taken place (54–56). Although this process of demographic recovery of the central city has occurred in Barcelona later (2000, turning point) than what happened in other world metropolises, such as London (1980) or New York (1980). In this context of demographic change, the increase in the foreign population has been especially significant, going from representing 46,015 people in 2000 (3.07% of the city's population) to 457,245 people in 2025 (26.4%).

The aging of the population is a proven fact in the city of Barcelona. The number of births has been steadily declining, reaching one-third in 2024 compared to 1975 (Figure 3b). In 2023, the city reached its all-time low (11,378 births, compared to 33,773 in 1975).^7^

Older adults (>= 65 years) have continued to increase in the period 1997–2025, far exceeding the child-age population (< = 14 years). Between 2010 and 2025 the difference between the two age groups has been steadily increasing, going from 137,000 in 2010 to nearly 170,000 in 2025 (see Figure 3c).

However, the explosion of the immigrant population masks the aging process of Barcelona's population. The continued influx of foreign population has determined, as has been seen, the increase in the total population of Barcelona, which has meant that the proportion of the older adults has not changed practically throughout the entire period 1997–2025, remaining at about 21% of the total of the city. If we only consider the proportion of older people in relation to the population of Barcelona (right axis in Figure 3d), it would seem that the city is not experiencing a marked aging process. However, if we compare it to the total population aged 65 and over (left axis), a clear demographic aging becomes apparent.

The aging population has a very marked gender component in Barcelona. The greater life expectancy of women (57) means that not only there are more older adults women than men, but that this difference becomes increasingly pronounced from the age of 80 onwards. In 2022, life expectancy at birth for women was 86.9 years, compared to 81.3 years for men, although this difference has been decreasing over the last 25 years, going from 7.3 in 2000 to the current 5.6 years (58). In 2025, in the 80 and over age group, women represented 69.74%, compared to only 57.21% of people between 65 and 79 years old. The feminization of old age is, therefore, an undeniable fact in the city of Barcelona, especially among the older age groups.

**Figure 4:**
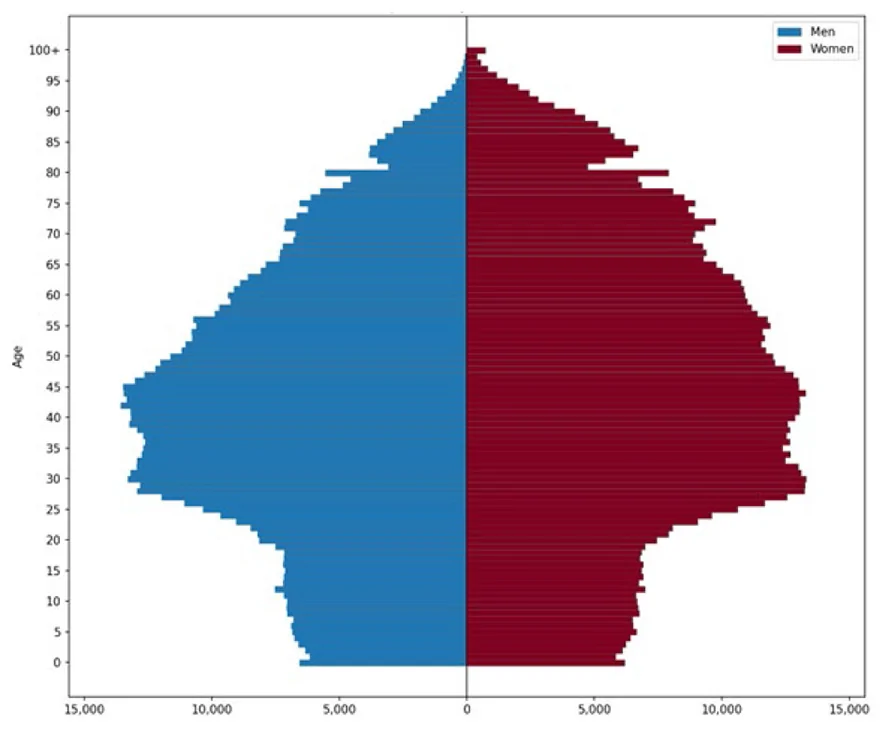
Age pyramid. Barcelona 2021.

The demographic trend, in the short term, is toward a significant increase in the older adults population. The age pyramid (Figure 4) shows how the 40–49 age group, which was the largest in 2021, will reach or exceed 65 years of age by 2050, representing a potential increase of 260,000 older people in that year.

**Figure 5:**
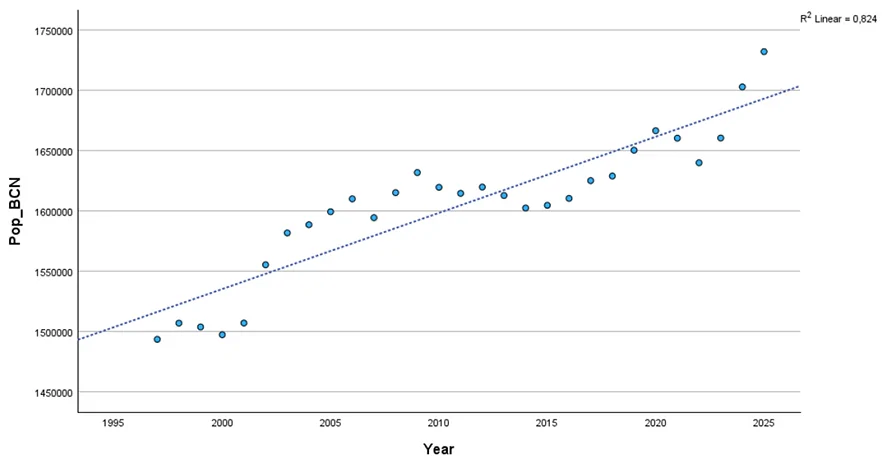
Population vs. Year (1997–2025).

The forecasts made by IDESCAT (Institut de Estadístic de Catalunya) for the period 2024–2044 help to estimate the older adults population of Barcelona in the target year (2050). In this regard, it should be noted that of the three scenarios projected by IDESCAT (high, medium, and low), the high scenario appears to be the one that best reflects current demographic trends. The OLS model of Barcelona's population growth between 1997 and 2025 (Figure 5) forecasts 1,850,914 inhabitants by 2050, relatively close to the high scenario estimate in IDESCAT's 2024 projection.

Following this high scenario, by 2050 Barcelona is expected to reach more than 490,000 people aged 65 years or older. One in four city residents will be of advanced age.

Given the current situation of the older adults population, as well as the result of the most likely projection, it is important to emphasize the need for the city of the older adults to adapt to climate change.

### 3.2 Global warming and heat waves

**Figure 6:**
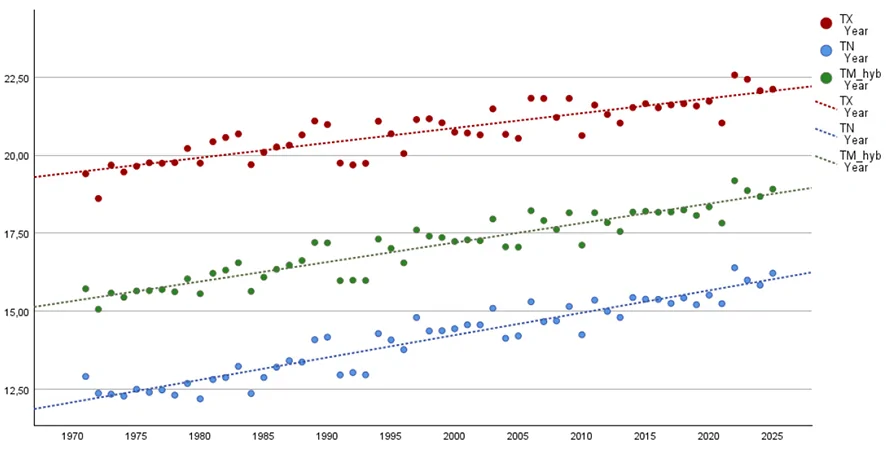
Evolution of TM, TX and TG (1971-2025).

Barcelona's climate has been experiencing a continuous warming process in recent decades. Taking El Raval station as a reference, average temperatures (TM) have risen 3.35°C between 1971 and 2025,^8^ more than the whole of Spain and the Mediterranean region.^9^ The increase is more pronounced in minimum temperatures (TN), 3.88°C, than in maximum temperatures (TX), 2.59°C. Without a doubt, Barcelona is a hotspot in the context of global warming. Figure 6 shows the temporal evolution of TM, TX & TN.

**Figure 7:**
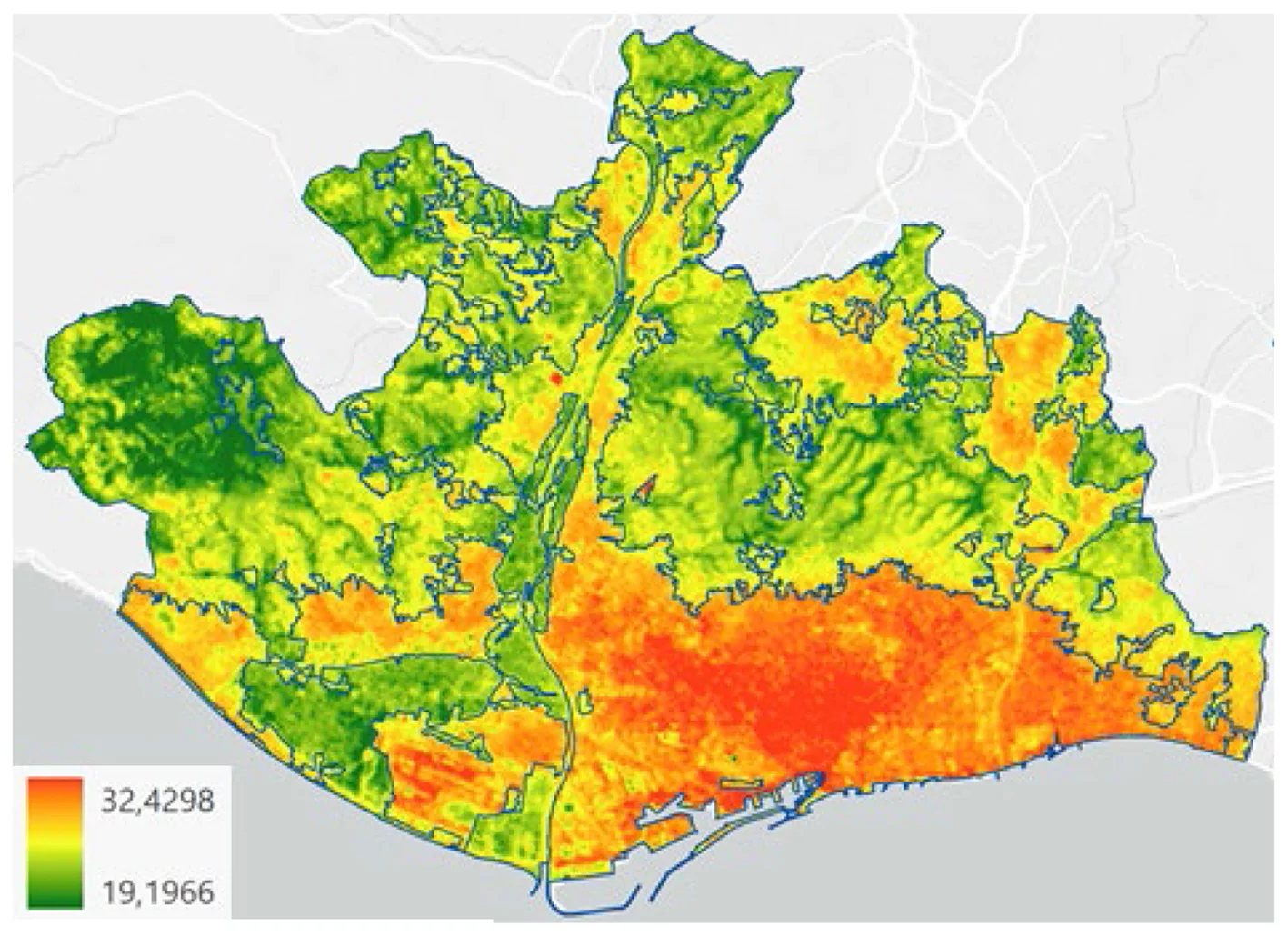
LST Barcelona Metropolitan Area (08-15-2025). Source: Arellano and Roca (65).

The urban heat Island (UHI) is undoubtedly a factor that determines the greater increase in temperature in Barcelona (59–61), in relation to the rest of peninsular Spain, as well as the western Mediterranean region. In the case of Barcelona, on the shores of the Mediterranean Sea, the UHI is especially pronounced at night. The sea, which has a beneficial effect during the day by moderating maximum temperatures, does not exert a beneficial effect during summer nights. The daytime breeze (“marinada”) blows from the sea to the land, cooling the air. However, during summer nights there is practically no air movement (the “terral,” from land to sea), hindering the dissipation of the heat accumulated during the day. Figure 7 shows the spatial distribution of the nocturnal LST of the Barcelona metropolitan area during a typical nocturnal heat wave (15-08-2025). The average LST of urban land (in blue in the figure, 27.4°C) is 2.3°C higher than that of rural land (25.10°C).

The 95th percentiles of the hottest days and nights of the period 1971–2000, following the definition of AEMET (30, 33) used for the determination of heat waves, are established at 30.9°C (TX) and 23.8°C (TN). If maximum or minimum temperatures exceed these thresholds for 3 or more consecutive days (DHW) or nights (NHW) heat waves are identified.

Between 1971 and 2025, 524 days have exceeded the TX threshold and 803 nights have exceeded the TN threshold. And a total of 54 DHW and 93 NHW have been produced.

Table 3 shows the evolution of the number of DHW & NHW by decade (weighted by the number of years). As can be seen, the number of heat waves is clearly increasing. If we look at DHWs (Daytime Heat Waves), these have risen from 1 to 15 per decade between 1971 and 2025. Even more outstanding is the increase in NHW (nighttime heat waves), which have gone from 0 in the first two decades to 36 in the period 2011–2020 (27 between 2021 and 2025). The duration of heat waves shows a clear increasing trend. The average duration of DHW has increased from 3 days in the decade 1971–1980 to 6.07 days in the period 2021–2025. Meanwhile, NHWs have gone from 0 nights (1971–1990) to 8.63 nights (2021–2025).

**Table 3:** Number and duration of HW by decade.

Type	Decade	Number HW	Number of days	Number of days by HW
DHW	1971–1980	1	3	3.00
DHW	1981–1990	5	20	4.00
DHW	1991–2000	4	16	4.00
DHW	2001–2010	14	81	5.79
DHW	2011–2020	15	62	4.13
DHW	2021–2025	15	91	6.07
NHW	1991–2000	9	39	4.33
NHW	2001–2010	21	137	6.52
NHW	2011–2020	36	211	5.86
NHW	2021–2025	27	233	8.63

The warming process experienced in Barcelona is consistent with the GW process on a Mediterranean scale (“extremely likely”). The projection to the near future (2050), using the widely validated empirical methodology of KTS regression and Sen's slope, confirms the significance of the warming of the city and its metropolitan area. Table 4 shows the basic parameters of the trend in average, maximum, and minimum temperatures, as well as their projection to 2050.

**Table 4:** Temperature projection (2050).

Variable	Kendall tau	*p*-value (MK test)	KTS slope (°C/year)	KTS intercept (°C)	1971	2025	2050	2025–1971	2050–2025
TX	0.680808	2.15E-13	0.047437	−74.070	19.43	21.99	23.18	2.56	1.19
TN	0.80202	5.33E-18	0.071187	−127.99	12.32	16.16	17.94	3.84	1.78
TM	0.762963	1.95E-16	0.06212	−106.917	15.52	18.88	20.43	3.35	1.55

The Mann-Kendall (MK) test as well as Kendall's tau show the very high statistical confidence of the trend experienced by the temperatures.^10^ The temperature projection to 2050 confirms the hypothesis of a greater increase in minimum temperatures (1.78°C), compared to the mean (1.55°C) and, above all, the maximum temperatures (1.19°C) compared to 2025. By 2050, the average annual temperature will exceed 20°C, almost 5°C higher than in 1971.

Extreme temperature events will follow a similar pattern. Table 5 shows the trends related to daytime and nighttime heat waves, as well as the duration of days and nights of extreme heat.

**Table 5:** Frequency and duration of HW.

Indicator	Kendall tau	*p*-value (MK test)	KTS slope	KTS intercept	2025	2050	2025–1971	2050–2025
DHW frequency (events/year)	0.530880	4.78E-07	0.03125	−61.4219	1.86	2.64	1.69	0.78
NHW frequency (events/year)	0.694191	1.05E-11	0.083333	−165.458	3.29	5.38	4.5	2.08
DHW duration (total days/year)	0.503275	6.17E-07	0.148148	−292.926	7.07	10.78	8	3.7
NHW duration (total days/year)	0.707592	1.62E-12	0.589744	−1175.01	19.22	33.96	31.85	14.74

The KTS models predict an increase of one DHW per year, as well as two additional NHWs, in 2050 compared to 2025. It also increases the duration of DHWs by almost 4 days and NHWs by 15 nights. More frequent and longer-lasting heat waves in 2050. Especially during nighttime heat waves. By 2050, these will be twice as frequent as daytime heat waves, and the duration of nighttime heat waves will be three times longer than that of daytime heat waves. More than a month of extreme nighttime heat!

### 3.3 High temperatures and mortality

**Figure 8:**
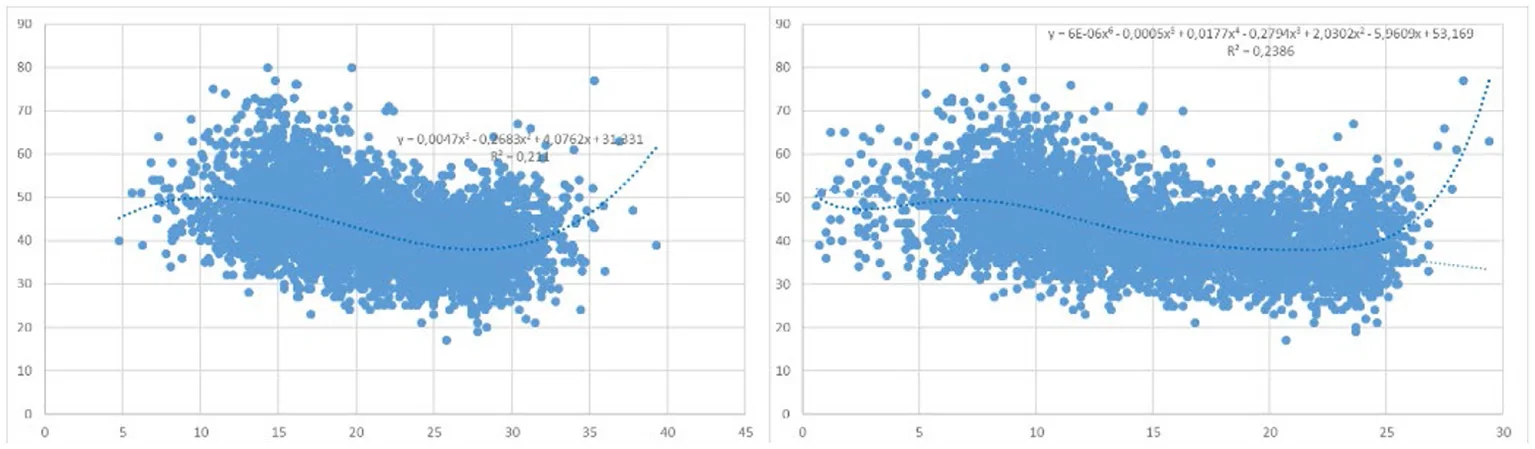
Deaths vs. TX and TN (2007–2018).

The effect of high temperatures on health can be estimated based on mortality rates throughout the year. Figure 8 shows the relationship with TX and TN, between January 1, 2007 and December 31, 2018. The fit of a curvilinear 6-degree model shows how mortality gradually decreases with increasing temperatures, but that from a maximum temperature of 26.9°C, and a minimum temperature of 21.3°C, this process is reversed, increasing the number of deaths.

The higher mortality rate during winter, resulting from low temperatures and the consequent increase in winter illnesses (flu, COVID, etc.), tends to decrease with the warmer temperatures of spring and early summer (62, 63). However, this reduction in mortality is halted by the arrival of extreme temperatures. Heat waves (both day time and night time) significantly increase mortality, especially among the most vulnerable.

**Figure 9:**
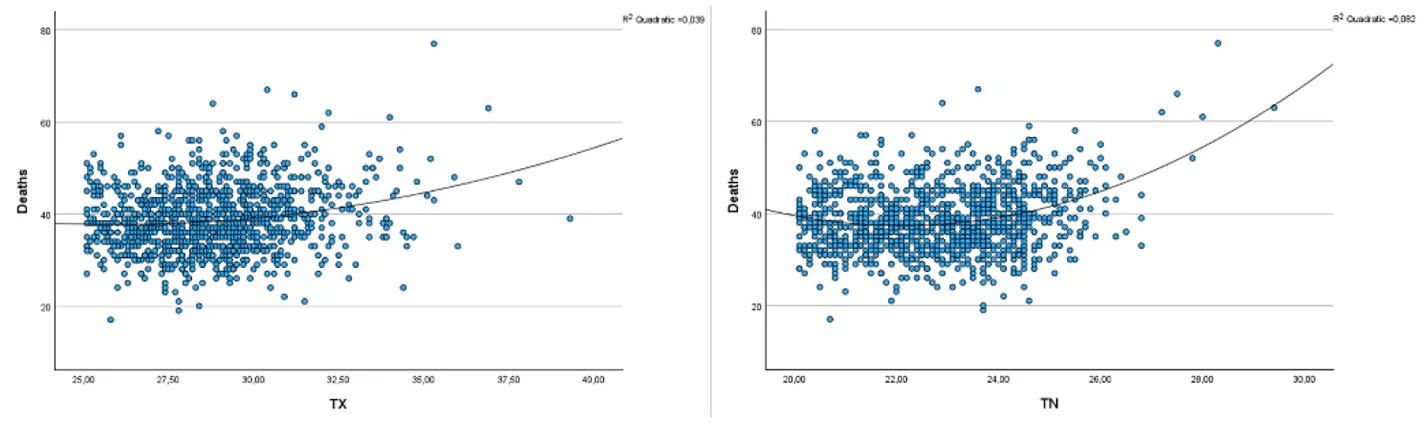
Deaths vs. TX (> 25 °C) & TN (> 20 °C).

In order to analyse the effect of summer temperatures on mortality, the days in which temperatures exceed 25 °C TX (“summer days” according to Copernicus) and 20°C TN (“tropical nights”) are selected. Figure 9 shows the effect of high temperatures on mortality.

The quadratic model shows a markedly higher correlation (R^2^ = 0.082) between observed mortality and TN. TX denotes a much lower incidence (R^2^ = 0.039) in daily deaths. The slope of the aforementioned quadratic models (obtained from their derivative) confirms the much greater impact of minimum temperatures (slope = 0.984) than maximum temperatures (slope = 0.169) in determining mortality in severe heat events.

Despite what is commonly asserted in academic literature (17, 44, 64), minimum temperatures therefore appear to be more decisive than maximum temperatures in explaining mortality in the city of Barcelona.

**Figure 10:**
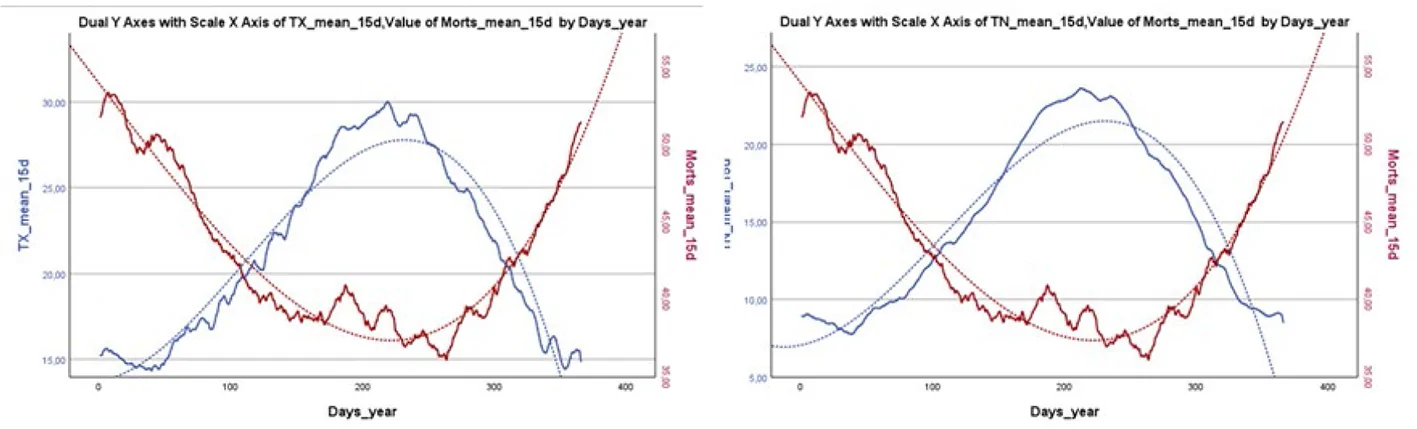
Deaths vs. TX & TN (15 days mean).

In order to smooth out the variation in data relating to deaths and maximum and minimum temperatures, averages were calculated for a 15-day period around each calendar day (from lag−7 to lag +7), as well as for the entire period (2007–2018). Figure 10 shows the evolution of mortality throughout the year in relation to temperature variations. As can be seen, the annual temperature pattern is clearly the opposite of that of mortality. As temperatures rise, mortality decreases. Furthermore, fitting a cubic model (as opposed to a quadratic one) allows us to observe the more gradual decline in deaths until reaching their minimum, compared to the more pronounced increase toward the end of the year.

**Figure 11:**
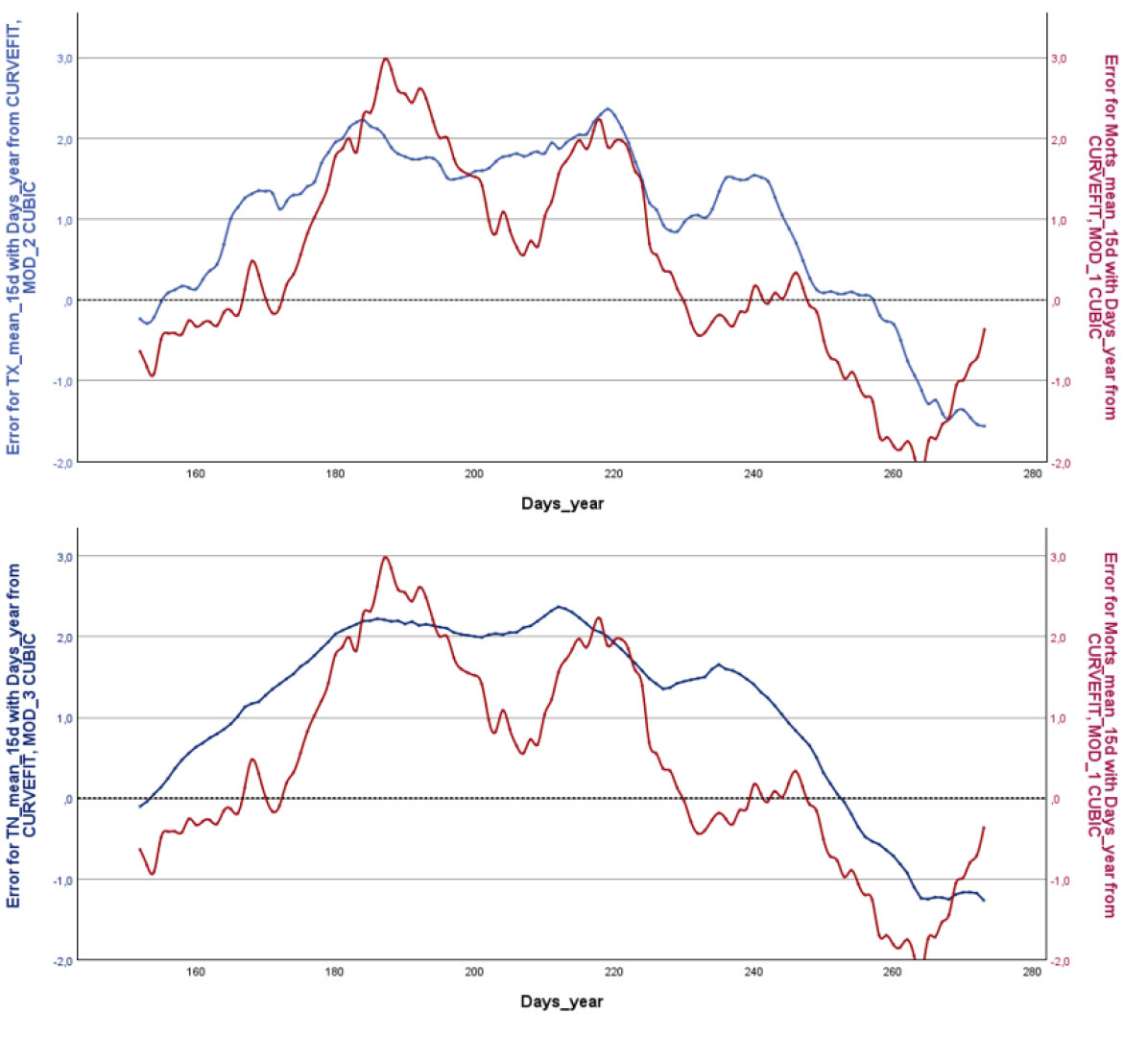
Temporal distribution of errors in the cubic models of TX, TN & Deaths (summer).

Fitting the cubic model of deaths over the 365 days of the year allows us to identify the reference mortality for each calendar day and, consequently, to detect the days of the year where mortality is higher than expected. Figure 11 shows the temporal distribution of the errors of this cubic model (referring to daily deaths) throughout the summer (June to September). The fact that these errors show a positive sign indicates a higher observed mortality than expected, confirming the summer excess mortality due to the heat. Comparison with the residuals of the cubic models of maximum and minimum temperatures shows that this additional mortality is due to the extreme temperatures experienced throughout the summer.^11^

The non-linear model fit between the residuals resulting from the cubic model of deaths (dependent variable) and maximum and minimum temperatures (independent variables) confirms the positive impact of temperatures on summer excess mortality. Tables 6, 7 show these models for both TX and TN. During the summer, higher temperatures are associated with higher mortality (unlike what occurs during the rest of the year).

Table 6Statistical residues of summer mortality vs. maximum temperatures (cubic model).

**Table:** 

Model summary				
Model	R	R Square	Adjusted R square	Std. error of the estimate
1	0.626ª	0.392	0.387	0.9937921

**Table:** 

Coefficients						
Model	Predictor	B	Std. Error	Beta	t	Sig.
1	(Constant)	−13.254	1.557		−8.512	<0.001
	TX_mean_15d	0.493	0.056	0.626	8.788	<0.001

ªPredictors: (Constant), TX_mean_15d. Dependent Variable: Error for Morts_mean_15d with Days_year from CURVEFIT, MOD_1 CUBIC.

Table 7Statistical residues of summer mortality vs. minimum temperatures (cubic model).

**Table:** 

Model summary				
Model	R	R Square	Adjusted R Square	Std. Error of the Estimate
1	0.675ª	0.455	0.451	0.94023436

**Table:** 

Coefficients						
Model	Predictor	B	Std. error	Beta	t	Sig.
1	(Constant)	−10.819	1.124		−9.627	<0.001
1	TN_mean_15d	0.520	0.052	0.675	10.017	<0.001

ªPredictors: (Constant), TN_mean_15d. Dependent Variable: Error for Morts_mean_15d with Days_year from CURVEFIT, MOD_1 CUBIC.

The higher R^2^ (and the higher regression coefficient) of the model with TN as the independent variable confirms the hypothesis regarding the greater impact of minimum temperatures on summer mortality. While higher temperatures (maximum or minimum) are associated with higher mortality, the impact of TN is greater than that of TX.

In addition to estimating meteorological heat waves (HW), health heat waves (HHW) can be established, distinguishing between daytime (DHHW) and nighttime (NHHW). In this study, and as explained in the section on Methodology, the association between temperature and daily mortality was modeled using generalized additive models (GAMs) with a Poisson distribution and a log link function, appropriate for count data.

Taking the 50th percentile (P50) of the probability of temperatures as a reference for the model (TX = 27.8°C; TN = 21.9°C), the results have been obtained for RR = 1.05 (moderate risk), 1.1 (high risk) and 1.15 (extreme risk). In order to eliminate any potential variations in RR, the aforementioned thresholds were obtained consistently (0.5°C, 1°C).^12^ This removes sensitivity to local oscillations in the GAM curve. The attached Table 8 shows these thresholds.

**Table 8:** Thresholds used to define extreme heat events that affect health (1 day).

Exposure	RR_target	Tref_P50	Threshold_simple	Threshold_ sustained_0p5C	Threshold_ sustained_1p0C
TX	1.05	27.8	32.2	32.2	32.2
TX	1.1	27.8	34.3	34.3	34.3
TX	1.15	27.8	35.9	35.9	35.9
TN	1.05	21.9	24.4	24.4	24.4
TN	1.1	21.9	25.4	25.4	25.4
TN	1.15	21.9	26	26	26

Initially, the threshold relative to RR = 1.1 will be used for the definition of extreme heat that affects health. The TX threshold (34.3°C) corresponds to P98. For its part, the TN threshold (25.4°C) corresponds to P97. It should be added that the 95th percentile for TX is reached at 32.1°C, as well as 25.1°C for TN.

To define a heat wave, in addition to exceeding a certain temperature threshold, a certain degree of persistence is required. Prolonged heat affects health more than a single, isolated event that does not last over time. In this study, we have used as a reference point a heat wave lasting ≥ 2 days for TX (or 2 nights for TN), ≥ 3 days, and ≥ 4 nights. The primary reference point will be a heat wave lasting 3 days (DHHW) or 3 nights (NHHW). Table 9 shows the total number of health heat waves, as well as their duration, due to high maximum (DHHW) and minimum (NHHW) temperatures. It also includes the combination of both events, as well as their intersection.

**Table 9:** Health heat waves events.

Definition	k_min_days	n_episodes	total_episode_days
DHHW (TX>= 34.3C)	2	5	14
NHHW (TN>= 25.4C)	2	9	38
HHW union (TX or TN)	2	10	41
HHW intersection (TX and TN)	2	4	11
DHHW (TX>= 34.3C)	**3**	**4**	**12**
NHHW (TN>= 25.4C)	**3**	**7**	**34**
HHW union (TX or TN)	**3**	**8**	**37**
HHW intersection (TX and TN)	**3**	**3**	**9**
DHHW (TX>= 34.3C)	4	0	0
NHHW (TN>= 25.4C)	4	4	25
HHW union (TX or TN)	4	4	25
HHW intersection (TX and TN)	4	0	0

The above approach, heat-mortality relationships were explored using temperature-specific daily relative risks (RR_1d_), leading to high apparent thresholds for both daytime and nighttime temperatures (TX ≈ 34.3 °C; TN ≈ 25.4 °C for RR = 1.10). These values correspond to extreme daily temperatures associated with elevated mortality risk on individual days, and are consistent with the upper tail of the exposure–response function. These thresholds and the associated events are statistically valid and describe high-severity heat episodes, characterized by days that are individually associated with markedly elevated mortality risk.

However, this definition does not fully align with the operational logic of heat–health warning systems, which are designed to capture cumulative health risk arising from sustained exposure, rather than from isolated daily extremes. Heat–health warning systems and public-health interventions are not designed to respond to isolated daily extremes, but rather to persistent heat events. Consequently, defining heat-health waves on the basis of daily relative risk alone may substantially overestimate the temperature thresholds relevant for real-world health impacts.

For this reason, the analytical focus was subsequently shifted toward an episodic risk framework, in which heat-health waves are defined by the persistence of elevated temperatures over at least three consecutive days (k ≥ 3). Within this framework, thresholds were recalibrated to achieve an equivalent episodic relative risk (RR_3d_ = 1.10). This recalibration yields substantially lower thresholds (TX ≈ 30.2°C; TN ≈ 24.0°C), which better represent the onset of clinically relevant heat-health impacts and allow direct comparison with official operational thresholds. These thresholds correspond to P83 in the case of TX and P82 for TN (lower extremes than those obtained previously).

The preceding analysis allows us to estimate that 571 deaths were attributed to daytime heat waves (DHHW) during the study period (2007–2018), and 721 deaths were attributed to nighttime heat waves (NHHW). This also includes 15 DHHW episodes and 34 NHHW episodes during the same period.

Importantly, this shift does not reflect a change in the underlying temperature–mortality relationship, but rather a change in the epidemiological object of interest: from isolated daily risk (RR_1d_) to cumulative risk during persistent heat exposure (RR_3d_). The latter provides a more appropriate basis for defining heat-health waves and for comparison with official heat-warning thresholds.

Together, these two perspectives distinguish between rare, high-intensity heat episodes and more frequent, health-relevant heat-health waves, providing a coherent framework for both etiological analysis and public-health application.

These results highlight the importance of explicitly accounting for event duration when translating exposure–response relationships into operational heat-health thresholds, and demonstrate that nocturnal temperatures reach equivalent health risk levels at substantially lower absolute values than day time temperatures.

Both analyses confirm, once again, that in Barcelona, the heat waves associated with higher mortality rates are those caused by high nighttime temperatures. Nighttime heat waves (NHHW) are more frequent and last longer than nighttime heat waves (DHHW). The official rhetoric, which focuses solely, or primarily, on maximum temperatures, breaks down sharply in Barcelona.

### 3.4 Mortality among the older adults due to high temperatures

As noted in the previous section, mortality in Barcelona increases with high daytime and nighttime temperatures. This mortality primarily affects the most vulnerable groups, such as the older adults. Unfortunately, the analysis of the relationship between mortality and temperatures carried out in the previous section (2007–2018) cannot be replicated by age group. Daily mortality data, broken down by age group, is only available for the province of Barcelona, not for the municipality. So, due to the lack of age-disaggregated daily mortality data at the city level, the main analysis was conducted at the provincial scale, which provides reliable daily mortality by age group and captures the same climatic exposure affecting the metropolitan area of Barcelona.^13^ The provincial analysis estimates incremental temperature–mortality associations under heterogeneous climatic conditions and does not define health thresholds, which were addressed at the city scale.

As indicated in the methodology section, daily deaths (https://momo.isciii.es/panel_momo/) as well as maximum and minimum temperatures (ERA5) were analyzed between January 1, 2015 and December 31, 2025:

- The analysis of the effects of high daytime and nighttime temperatures (TX & TN) on mortality was limited to the summer months (June to September). The year 2020 was excluded as a precaution due to the COVID-19 pandemic.
- A Poisson model (GLM) was developed, in which daily mortality of the older adults (or of the population as a whole) is the dependent variable, and temperature (TX or TN) is the independent variable. Considering the seasonality of the data, as well as its potential influence from the day of the week on which it was recorded, the variables time and DOW were included as adjustment factors. So, daily counts of deaths were analyzed using Poisson regression models with a log link, adjusting for long-term trend (time) and day of the week (DOW).

Tables 10, 11 show the results of the models of daily deaths among the older adults as a function of maximum and minimum temperatures.

Table 10Model older adults deaths vs. TX (Poisson model).

**Table:** 

Generalized linear model regression results			
Dep. Variable:	Deaths_elderly	No. Observations:	1220
Model:	GLM	Df Residuals:	1211
Model Family:	Poisson	Df Model:	8
Link Function:	Log	Scale:	1.0000
Method:	IRLS	Log-Likelihood:	−5040.4
Date:	Fri, 23 Jan 2026	Deviance:	2174.6
Time:	18:00:50	Pearson chi2:	2.20e+03
No. Iterations:	4	Pseudo R-squ. (CS):	0.4169
Covariance Type:	nonrobust

**Table:** 

Parameter estimates						
Variable	coef	std err	z	*P* > |z|	[0.025	0.975]
const	4.1380	0.027	153.913	0.000	4.085	4.191
TX	0.0155	0.001	15.988	0.000	0.014	0.017
time	9.486e-05	7.97e-06	11.909	0.000	7.92e-05	0.000
1	0.0623	0.011	5.880	0.000	0.042	0.083
2	0.0722	0.011	6.840	0.000	0.051	0.093
3	0.0894	0.010	8.520	0.000	0.069	0.110
4	0.1003	0.010	9.594	0.000	0.080	0.121
5	0.0529	0.011	5.002	0.000	0.032	0.074
6	−0.0321	0.011	−2.967	0.003	−0.053	−0.011

TX beta (Deaths_elderly): 0.015455673020141981. TX RR per +1°C (Deaths_elderly): 1.0155757296556405.

Table 11Older adults vs. TN (Poisson model).

**Table:** 

Generalized linear model regression results			
Dep. Variable:	Deaths_elderly	No. Observations:	1220
Model:	GLM	Df Residuals:	1211
Model Family:	Poisson	Df Model:	8
Link Function:	Log	Scale:	1.0000
Method:	IRLS	Log-Likelihood:	−5065.4
Date:	Fri, 23 Jan 2026	Deviance:	2224.7
Time:	17:53:18	Pearson chi2:	2.26e+03
No. Iterations:	4	Pseudo R-squ. (CS):	0.3924
Covariance Type:	nonrobust

**Table:** 

Parameter estimates						
Variable	coef	std err	z	*P* > |z|	[0.025	0.975]
const	4.2225	0.024	174.898	0.000	4.175	4.270
TN	0.0170	0.001	14.315	0.000	0.015	0.019
time	8.814e-05	8.01e-06	11.004	0.000	7.24e-05	0.000
1	0.0579	0.011	5.472	0.000	0.037	0.079
2	0.0687	0.011	6.517	0.000	0.048	0.089
3	0.0885	0.010	8.434	0.000	0.068	0.109
4	0.1010	0.010	9.656	0.000	0.080	0.121
5	0.0499	0.011	4.714	0.000	0.029	0.071
6	−0.0328	0.011	−3.034	0.002	−0.054	−0.012

TN beta: 0.01697181793064057. TN RR per +1°C: 1.0171166574691377.

Models confirm the increase in mortality among the older adults with rising temperatures. One-degree Celsius increase in maximum temperature represents a 1.56% increase in the number of deaths. Similarly, an increase of 1 degree in the minimum temperature implies a 1.71% increase in the number of deaths. Both models are statistically significant (*p*-value < 0.001).

Despite the greater impact of the TN (reflected by the model coefficient), the pseudo-R^2^ of the TX (0.4169) exceeds that of the TN (0.3914), suggesting that, at the provincial level, maximum temperatures have an equal or greater impact than minimum temperatures. These results differ from those previously obtained in the city of Barcelona, where the minimum temperature plays a particularly significant role. The urban heat island effect at night has a decisive impact on health.

Tables 12, 13, meanwhile, show the results for the population as a whole. The models confirm the results obtained for people aged ≥ 65, although the incidence of temperatures is somewhat lower than in the older adults: an increase in the number of deaths of 1.015 and 1.016, respectively, for each degree of increase in TX and TN.

Table 12Total population vs. TX (Poisson model).

**Table:** 

Generalized linear model regression results			
Dep. Variable:	Deaths_tot	No. Observations:	1220
Model:	GLM	Df Residuals:	1211
Model Family:	Poisson	Df Model:	8
Link Function:	Log	Scale:	1.0000
Method:	IRLS	Log-Likelihood:	−5099.4
Date:	Fri, 23 Jan 2026	Deviance:	2095.8
Time:	17:35:33	Pearson chi2:	2.13e+03
No. Iterations:	4	Pseudo R-squ. (CS):	0.3901
Covariance Type:	nonrobust

**Table:** 

Parameter estimates						
Variable	coef	std err	z	*P* > |z|	[0.025	0.975]
const	4.3393	0.025	174.800	0.000	4.291	4.388
TX	0.0148	0.001	16.562	0.000	0.013	0.017
time	5.861e-05	7.35e-06	7.971	0.000	4.42e-05	7.3e-05
1	0.0596	0.010	6.095	0.000	0.040	0.079
2	0.0712	0.010	7.312	0.000	0.052	0.090
3	0.0840	0.010	8.666	0.000	0.065	0.103
4	0.0955	0.010	9.895	0.000	0.077	0.114
5	0.0516	0.010	5.284	0.000	0.032	0.071
6	−0.0291	0.010	−2.921	0.003	−0.049	−0.010

TX beta (Deaths_tot): 0.014791594855022488. TX RR per +1°C (Deaths_tot): 1.0149015318733408.

Table 13Total population vs TN (Poisson model).

**Table:** 

Generalized linear model regression results			
Dep. Variable:	Deaths_tot	No. Observations:	1220
Model:	GLM	Df Residuals:	1211
Model Family:	Poisson	Df Model:	8
Link Function:	Log	Scale:	1.0000
Method:	IRLS	Log-Likelihood:	−5129.7
Date:	Fri, 23 Jan 2026	Deviance:	2156.3
Time:	17:40:04	Pearson chi2:	2.19e+03
No. Iterations:	4	Pseudo R-squ. (CS):	0.3590
Covariance Type:	nonrobust

**Table:** 

Parameter estimates						
Variable	coef	std err	z	*P* > |z|	[0.025	0.975]
const	4.4252	0.022	198.742	0.000	4.382	4.469
TN	0.0160	0.001	14.600	0.000	0.014	0.018
time	5.241e-05	7.39e-06	7.088	0.000	3.79e-05	6.69e-05
1	0.0554	0.010	5.667	0.000	0.036	0.075
2	0.0679	0.010	6.971	0.000	0.049	0.087
3	0.0830	0.010	8.569	0.000	0.064	0.102
4	0.0961	0.010	9.950	0.000	0.077	0.115
5	0.0486	0.010	4.977	0.000	0.029	0.068
6	−0.0298	0.010	−2.991	0.003	−0.049	−0.010

TN beta (Deaths_tot): 0.015974535403052915. TN RR per +1°C (Deaths_tot): 1.016102810428124.

The provincial study, therefore, confirms the negative effect of increased maximum and minimum temperatures on health, especially on the health of the older adults. Nighttime temperatures also tend to have a greater effect than daytime temperatures. However, their effect is attenuated, as will be discussed in section 4.4 of this article.

## 4 Discussion

### 4.1 Barcelona, an increasingly aging city progressive aging

The progressive aging of Barcelona's population is an undeniable fact, even though the proportion of older adults people remains relatively constant at around 21%, due to the continuous influx of immigrants. The population over 64 years of age has continued to grow, going from 338,446 in 2011 to 361,914 in 2025. Of the 20 most populated Spanish cities in 2025, Barcelona is the one that has experienced an increase in population ≥65 compared to 2011.^14^

In this work, following the (high scenery) projection of IDESCAT, we have considered that the older adults will exceed at least 490,000 in 2050. The baby boomer generations,^15^ who are currently between 40 and 50 years old, will have reached 65 years of age or older by 2050, which represents a potential increase of 255,908 older people by that date.

**Figure 12:**
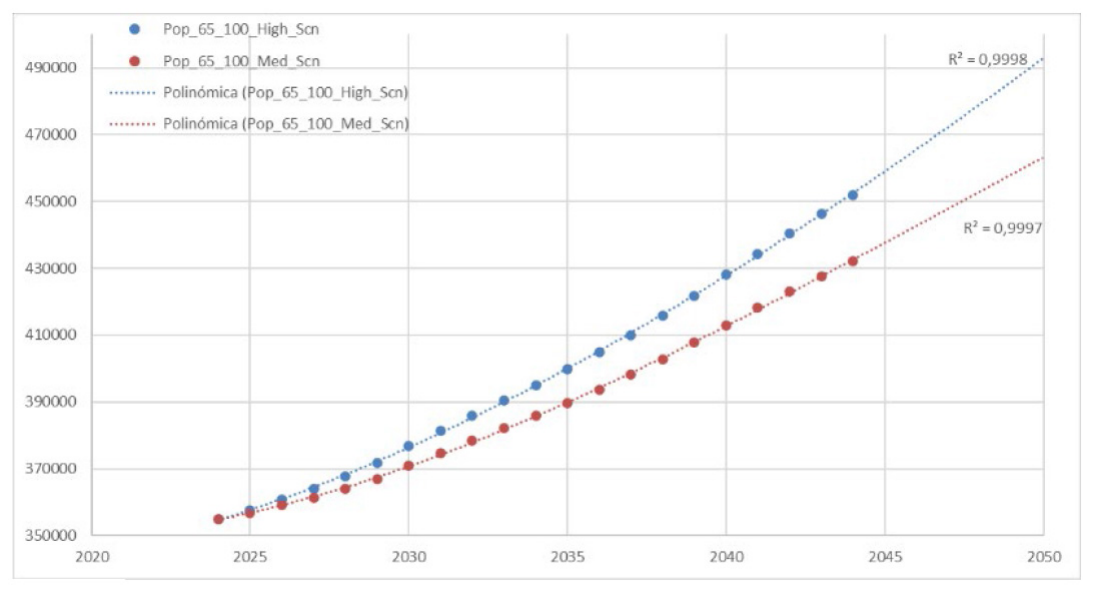
Medium & High Scenarios (IDESCAT, 2024–2044).

Figure 12 shows the projection to 2050 of the medium and high scenarios of IDESCAT (2024–2044). The aforementioned estimate of 490,000 people aged 65 and over by 2050 is likely a conservative estimate (despite being the high-end scenario). It probably presupposes an exaggerated emigration of cohorts aged 40–44 and older.^16^

Table 14 presents the evolution of the population of the cohorts that in 2050 will have a population of 65 years or older. The table above was calculated assuming no migration and that the current mortality rate for each age group remains constant. This means that all individuals aged 40 and over in 2025 (the cohorts who will be 65 or older in 2050) will remain in the city in that year. It also assumes that mortality rates will remain stable over time. Each step in the table (up to 5, equivalent to 2050) represents a change in the older adults population.

**Table 14:** Estimated population ≥ 65 years.

Cohort	Population	Step 1	Step 2	Step 3	Step 4	Step 5
2025	2025	(2030)	(2035)	(2040)	(2045)	(2050)
40–44	126,363	126,250	126,049	125,706	125,139	**124,342**
45–49	129,545	129,338	128,994	128,415	127,442	**126,301**
50–54	121,482	121,158	120,672	119,750	118,010	**116,888**
55–59	110,220	109,728	108,945	107,510	105,027	**103,835**
60–64	101,866	101,141	99,793	97,279	93,062	**92,287**
65–69	90,019	89,048	87,408	84,533	78,909	71,949
70–74	76,111	74,724	72,260	67,440	58,788	48,838
75–79	72,742	70,355	65,642	57,189	42,966	27,028
>=80	56,042	52,258	45,495	34,134	0	0
>=65	**294,914**	**387,527**	**479,544**	**567,835**	**624,204**	**711,467**

By 2050, if there was no migration and mortality rates remain at current levels, Barcelona could have 711,467 older adults residents. Therefore, IDESCAT's estimate of 490,000 older adults residents is likely an underestimate. Barcelona will undoubtedly be a city with an aging population, a population highly vulnerable to extreme temperatures.

### 4.2 Barcelona, hotspot of nighttime heat

**Figure 13:**
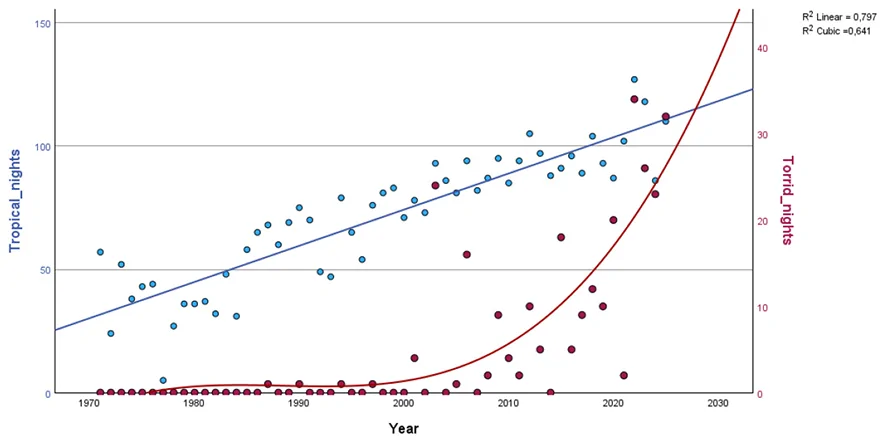
“Tropical” and “Torrid” nights (1971–2025).

Unlike the rest of the Iberian Peninsula (65) where maximum temperature has increased more than minimum ones, in Barcelona it is the minimum temperatures (an increase, between 1971 and 2025, of 3.88°C for TN compared to 3.35°C for TX), especially during the summer, that are experiencing a more pronounced increase. The warming of the Mediterranean Sea is the main cause. This process of nighttime warming during the summer is clearly observed in the evolution experienced in “tropical” nights (TN > 20 C) and “torrid” nights (TN > 25 C) (Figure 13).

As can be seen, while “tropical” nights have had a linear increase (R^2^ = 0.797, between 1971–2025), “torrid” nights have tended to grow exponentially (R^2^ = 0,641, cubic model). Looking at the last 25 years,^17^ the cubic model for “hot” nights reaches an R^2^ of 0.651, compared to 0.508 for the linear model of “tropical” nights. This shows a clear acceleration of the most extreme (“torrid”) temperatures.

This process of nighttime warming can be summarized by the following indicators:

- The average summer temperature of the TN has gone from 18.15°C in 1971 to 22.66°C in 2025, an increase of 4.5°C.^18^ A much greater increase than that experienced by the TX (2.92°C).^19^
- Since 2021, the number of nights with “tropical” temperatures has exceeded three and a half months (108.6 nights on average per year). Virtually all summer nights are “tropical.”
- Regarding the “torrid” nights, 2022 and 2025 exceeded one month in duration (34 and 32 days, respectively). Five of the six years with the most “torrid” nights (with the exception of 2003) have occurred since 2020, demonstrating the acceleration of nighttime warming in Barcelona.
- All of this has led to an extraordinary increase in the number and duration of nighttime heat waves. Between 2021 and 2025, there were 5.4 nighttime heatwaves (NHW) per year, compared to 3 daytime heatwaves (DHW). Furthermore, nighttime heatwaves lasted a total of 46.4 nights per year, exceeding the duration of DHW by 18.2 days.

Because heatwave occurrence exhibits a clear regime shift since the mid-1990s, trend analyses were repeated for a recent subperiod to better represent current dynamics. Trend lines derived from the full 1971–2025 period provide conservative estimates due to the absence of heatwave events in the early decades. When trends are recalculated for the recent 1995–2025 period, substantially steeper slopes emerge, indicating an accelerated intensification of both heatwave frequency and duration, particularly for nighttime heatwaves. Projections based on the full period provide conservative estimates, while those based on the recent period reflect ongoing intensification.

The trend in daytime heatwaves is more sensitive to individual extreme summers, such as 2003, resulting in higher interannual variability and wider confidence intervals. In contrast, nighttime heatwaves exhibit a more persistent and robust increase. Daytime heatwave trends exhibit higher interannual variability, partly influenced by exceptional summers such as 2003,^20^ which affected large parts of Western Europe simultaneously. This pan-European event contributes disproportionately to the daytime heatwaves, resulting in wider confidence intervals and a less robust trend estimate. In contrast, nighttime heatwaves display a more regular and persistent increase over time.

In conclusion, daytime heatwave trends are partly driven by large-scale, episodic extreme events in Europe, while nighttime heatwaves exhibit a more structural and persistent intensification. This distinction underscores the importance of separating daytime and nighttime heatwave processes when assessing long-term urban heat risk. Nighttime heat waves, therefore, represent the most extreme heat events in the city of Barcelona, suggesting the hypothesis supported in this work, regarding a greater effect on health compared to daytime heat waves.

### 4.3 Effect of maximum and minimum temperatures on health

One of the fundamental hypotheses of this article is that in Barcelona, minimum temperatures in summer are more decisive than maximum temperatures, if their effects on health (mortality) are considered. Contrary to the prevailing view, which considers that maximum temperatures have a dominant effect, in this work we have shown that in Barcelona, minimum temperatures are the most significant.

Several indicators show the predominance of minimum temperatures in summer mortality in the city of Barcelona:

- On the one hand, models relating to mortality in Barcelona on days when maximum temperatures relative to “summer days” (TX > 25°C) and nights with minimum temperatures above those of “tropical nights” (TN > 20°C) show a significantly stronger correlation between observed mortality and TN, compared to TX, which indicates a much lower incidence. The slope of the (quadratic) models confirms the much greater impact of TN (slope = 0.984) than TX (slope = 0.169) in determining mortality during severe heat events.
- The fit of the OLS model between the residuals resulting from the cubic fit between observed deaths and maximum and minimum temperatures (smoothed for the entire observed period) confirms the positive impact of temperatures on excess summer mortality. The higher R^2^ (and the higher regression coefficient) of the TN model confirms the hypothesis of this study regarding the greater impact of minimum temperatures on summer mortality. While higher temperatures (maximum or minimum) are associated with higher mortality, the impact of TN is greater than that of TX.
- But undoubtedly the element that determines the greatest effect of minimum temperatures is the study of the association between temperature (maximum or minimum) and daily mortality using a generalized additive model (GAM), adjusted using a Poisson distribution and a log link function. These models allow the establishment of thresholds, both daily and relative to three or more consecutive days, beyond which there is a high risk level (RR3d ≥ 1.1) of increased mortality. Exceeding the thresholds obtained by the GAM models for TX or TN, for this level of risk and persistence, has allowed for the identification of health-related heat waves (daytime and nighttime). These thresholds correspond to 30.2°C for maximum temperatures and 24.0°C for minimum temperatures, if they persist for three or more days, for the existence of a daytime (DHHW) or nighttime (NHHW) health-related heat wave.
- An additional factor that demonstrates the predominance of high nighttime temperatures over daytime temperatures in explaining mortality is that if you consider the daily risk (RR1d = 1.1), nighttime temperatures clearly dominate over daytime temperatures in explaining heat-related mortality. Using RR1d, health-relevant heat events derived from minimum temperatures are substantially more frequent and longer-lasting than those derived from maximum temperatures, resulting in a markedly higher number of attributable deaths.

The results obtained, in all the models analyzed, confirm that high minimum temperatures represent, in all scenarios, the main determining factor of the increase in summer mortality.

The results strongly contradict not only the majority state of the art in the literature, but specifically what is indicated in the official documents existing in Spain regarding the prevention of health risks from high temperatures. The National Plan of Preventive Actions against the effects of Excessive Temperatures on Health 2025, approved in April 2025, does not consider minimum summer temperatures as a determining element of risk (66). This Plan establishes low, medium, or high risk levels based on the sum of maximum temperatures exceeding the threshold for three consecutive days in each of the 182 weather-health zones into which Spain is divided.^21^

The comparison between the thresholds derived in this study and the official definition adopted by the Spanish Ministry of Health (ISCIII) highlights important conceptual and epidemiological differences for the case of Barcelona. For the littoral area of Barcelona, the ISCIII establishes a single threshold (TX ≥ 30.4°C for three or more consecutive days), not considering, as indicated, any reference to the TN. This threshold is not calibrated against the observed mortality risk. In contrast, the thresholds proposed in this paper for both daytime (TX) and nighttime (TN) heat waves are explicitly calibrated to achieve a predefined relative risk of mortality (RR_3d_ ≈ 1.10). While the ISCIII daytime threshold is numerically close to the RR-calibrated DHHW threshold identified in this study (30.2°C), the absence of a nocturnal criterion in the official framework leads to a substantial underestimation of the overall heat-related mortality burden.

**Figure 14:**
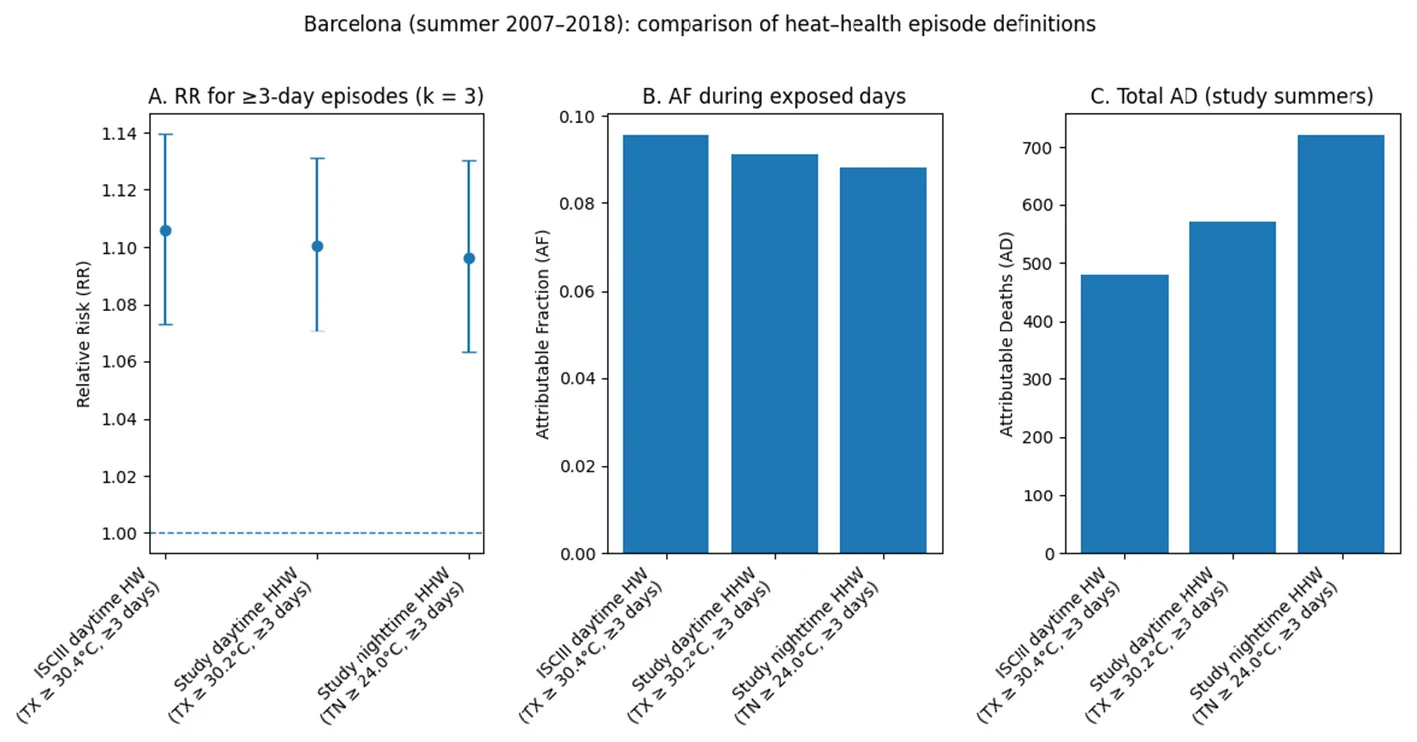
Comparison between the HHW obtained by TX, TN & ISCIII.

Figure 14 shows the relative risk (RR), attributable fraction, and deaths attributable to day and night health heat waves, in events of 3 or more days. In all the aforementioned indicators,^22^ the NHHW show greater sensitivity than the DHHW, confirming the hypothesis of the present research work. It is worth noting that the nighttime heat wave threshold (TN ≥ 24.0°C, k = 3) produces a relative risk comparable to the daytime threshold (RR_3d_ ≈ 1,1), but is associated with more frequent episodes, a greater number of days of exposure, and, above all, a much higher number of attributable deaths. These results demonstrate that nighttime heat is a determining factor in heat-related mortality in Barcelona, a dimension not reflected in current operational definitions based solely on daytime temperature.

Nighttime temperatures should therefore be included in the Spanish Government's “National Plan of Preventive Actions against the effects of Excessive Temperatures on Health.” Informing the public in advance of nighttime heat waves would increase resilience to extreme heat events, especially for the most vulnerable people.

In Catalonia, nighttime heat is taken into account in the citizen alert system. The Servei Meteorològic de Catalunya (SMC) develops an alert system for high temperatures which, in addition to using the maximums, also considers the minimums (https://www.meteo.cat/wpweb/divulgacio/la-prediccio-meteorologica/situacio-meteorologica-de-perill/) Specifically, for the Barcelona area it establishes the threshold of 34.1°C for TX and 26.5°C for TN (https://www.meteo.cat/wpweb/divulgacio/la-prediccio-meteorologica/situacio-meteorologica-de-perill/distribucio-territorial-de-llindars). The extreme heat threshold, according to the SMC, corresponds to the 98th percentile of the maximum or minimum temperature for June, July, and August over the last 10 years.^23^

However, the aforementioned thresholds are not adequate, reducing the capacity for reaction on the part of citizens. They are significantly higher, especially the nighttime threshold, which is why they don't seem to be truly useful for supporting a system of warnings to the public, such as heat alerts. 26.5 degrees, which corresponds to a low risk level according to the SMC, actually represents a risk level (RR) higher than 1.2. Well above high risk (1.1), even extreme (1.15). And the intermediate risk according to the SMT (27.5°C), actually corresponds to a very high RR = 1.35!! Furthermore, they only refer to the forecast of temperatures for the following day, when, as we have seen previously, the persistence of high temperature events is crucial.

Therefore, the SMC should also modify the high heat alarm system, especially at night, and consider the forecast not only for the next day, but for the following days.

### 4.4 Older adults mortality due to high temperatures

As indicated in section 3.4, daily mortality data for the city of Barcelona, broken down by age group, was not available. Only disaggregated daily mortality data for the province of Barcelona was obtained (https://momo.isciii.es/panel_momo/).

However, provincial data, despite being a useful reference, are not fully representative of the city of Barcelona. The population of the city of Barcelona represents only 28.7% of the provincial population (2024). As for the population over 64 years of age, Barcelona represents 30.9% of the province. In turn, Barcelona's temperatures, due to the urban heat island effect as well as its geographical location, are markedly higher than those of the province. This implies that modeling mortality as a function of temperature has a potentially substantial degree of error when extrapolated to Barcelona.

Provincial models confirm the increase in mortality among the older adults with rising temperatures. One degree Celsius increase in maximum temperature represents a 1.56% increase in the number of deaths. Similarly, an increase of 1 degree in the minimum temperature implies a 1.71% increase in the number of deaths. Although daily relative risks appear modest, their persistence over extended periods implies a substantial cumulative health burden, reinforcing the relevance of nighttime temperatures for public health. The provincial study therefore confirms the negative effect of the increase in maximum temperatures and, in particular, minimum temperatures on the health of older people.

However, the effect of high temperatures on mortality among those over 64 years of age is likely to be significantly higher in the city of Barcelona. Several factors support the above statement:

- Firstly, because the oldest population strata (≥ 80 years) are proportionally higher in Barcelona than in the province. Thus, the population aged ≥80 represents 34.5% of the total in the province, compared to a lower proportion when considering the ≥64 age group alone (30.9%). Because the older age groups have a larger proportion, the mortality rate may therefore be higher in Barcelona than that resulting from provincial data.
- The second factor contributing to a higher mortality rate among the older adults (and, in general, the population as a whole) relates to the city's location within the province. Because Barcelona is located on the Mediterranean coast, nighttime temperatures are high due to its proximity to the sea and, conversely, daytime highs (if altitude is not taken into account) are milder than in the rest of the province. And, since TN appears to play a particularly relevant role in heat-related mortality, the mortality rate in the city of Barcelona is likely underestimated.
- Barcelona tends to have higher minimum temperatures than its surrounding province, not only due to its proximity to the sea, but also due to the urban heat island effect (59, 60). Proportionally higher minimum temperatures lead to higher mortality, especially among the most vulnerable. Urban heat island effects not only elevate nighttime temperatures but also tend to trap air pollutants in the urban canopy layer, contributing to compound exposures that are harmful to older adults (67). The effect of nocturnal UHI, therefore, may further exacerbate mortality among the older adults.
- Finally, the temperature data (TX and TN) used, from ERA5, probably adjust the mortality models, smoothing out the extremes. ERA5 has difficulty capturing extremes, heat waves for example. And, due to its limited spatial resolution, the uniqueness of urban heat islands, such as that of Barcelona. For the reasons mentioned, the GLM models generated from ERA5 are likely to only partially reproduce the effects of temperatures on mortality in Barcelona.

Differences between the present results and previous city-level analyses may partly reflect the spatial scale and the temperature data source used. While observed urban measurements capture the full intensity and persistence of nocturnal heat island effects, ERA5 reanalysis data—particularly when spatially averaged at the provincial level—tend to smooth nocturnal temperature extremes. This attenuation of nighttime heat persistence may explain the weaker cumulative effects observed for minimum temperature compared with city-based studies

For the aforementioned reasons, the precise quantification of the effects of high temperatures, especially minimum temperatures, on the mortality of the older adults in Barcelona remains an outstanding issue.

## 5 Conclusions

This study provides robust evidence that high temperatures constitute a major and growing public health risk in Barcelona, particularly for older adults, in a context of accelerated global warming, pronounced nighttime warming, and rapid population aging. The results confirm that Barcelona is experiencing an exceptional increase in minimum temperatures, closely linked to its coastal location and urban heat island effect, leading to a marked intensification of nighttime heat waves.

By moving beyond purely meteorological definitions, this research proposes and operationalizes the concept of health heat waves (HHW), defined on the basis of epidemiologically derived temperature–mortality relationships and the persistence of heat exposure. Distinguishing between daytime (DHHW) and nighttime (NHHW) health heat waves reveals that nighttime heat waves are more frequent, longer-lasting, and associated with substantially higher mortality than daytime events. In Barcelona, nighttime temperatures reach equivalent health risk levels at lower absolute values than daytime temperatures, underscoring their critical role in shaping heat-related mortality.

The analysis demonstrates that minimum temperatures are more decisive than maximum temperatures in explaining summer mortality at the city scale, a finding that contrasts with the prevailing emphasis on daytime heat in official heat-warning frameworks. Provincial-scale models further confirm the negative impact of both maximum and minimum temperatures on older adults mortality, while suggesting that nighttime effects are likely underestimated due to spatial aggregation and the smoothing of extreme events in reanalysis data.

Taken together, these findings highlight the urgent need to revise current heat–health warning systems and prevention strategies, particularly by incorporating nighttime temperature thresholds and persistence criteria calibrated to health outcomes. As Barcelona continues to age and nighttime heat intensifies, addressing nocturnal thermal exposure emerges as a key priority for protecting vulnerable populations and enhancing urban climate resilience.

## Data Availability

The original contributions presented in the study are included in the article/supplementary material, further inquiries can be directed to the corresponding authors.

## Footnotes

1 For all estimates of temperature increases, heat waves, or other models considered in the article, they are not made taking into account the difference between the final year (e.g., 2024) and the initial year (e.g., 1971), but rather the estimates resulting from the corresponding OLS models.

2 Data relating to the increase in temperatures worldwide and in the Mediterranean region have been calculated from the average of the 30 models used by CMIP6. For Spain, CMIP6 calculates (as an average of the 30 reference models), 1.74°C, slightly below the Mediterranean region (1.83°C). These data differ significantly from those obtained by AEMET (1.96°C), which refer to the average of more than 1,500 meteorological stations (12).

3 With only a four-year difference (2020 and 2024), IDESCAT's projections have changed dramatically. If we analyze the “high” scenario, the estimated population for the entire city in 2044 changed from 1,732,137 (2020 projection) to 1,823,692 (2024 projection). That's almost 100,000 people! The 2020 projection failed spectacularly, at least in the short term, since it estimated a population of 1,658,537 inhabitants for 2025, when, in reality, 1,732,066 inhabitants were reached (January 1, 2025). Demographic projections, therefore, are far from being an exact science.

4 In the analysis of climate data, various regression methods have been employed to process datasets with diverse characteristics, particularly in the presence of outliers or non-linear trends. Ordinary least squares (OLS) regression is a commonly used parametric method for estimating linear relationships between variables. However, OLS is highly sensitive to outliers, which can result in biased estimates when it is applied to datasets with substantial variability or extreme values. Given that climate data often exhibit such complexities, more robust methods are required to ensure reliable trend estimation. To address these challenges, non-parametric methods like the Mann–Kendall test (MK) and Kendall–Theil–Sen (KTS) regression are frequently utilized. Unlike parametric approaches, the MK test and KTS regression do not assume any specific underlying distribution of the data (65).

5 In this study, the months of June through September, inclusive, have been considered “summer”. Although from a meteorological point of view AEMET considers “summer” only from June to August, the actual temperatures in the city of Barcelona suggest extending the warm period until September. During the study period (2007-2018), the average temperature in September in the city of Barcelona was almost identical to that of June (23.37 °C in June and 23.23 °C in September). If we consider TX, only the months of June and September exceeded an average of 25 degrees Celsius (26.67 °C in June and 26.19 °C in September), that is, the threshold corresponding to “summer days” according to Copernicus (TX > 25 °C). And only these same months exceeded, on average, the threshold of tropical nights (20.06 °C in June, and 20.27 °C in September). From a meteorological point of view, June and September are almost identical in the city of Barcelona, although, due to the process of adaptation to the heat, mortality is somewhat lower in September.

6 At the provincial level we have not used the same criteria to define the RRs defined in 2.3.3. because the provincial analysis aimed to estimate incremental temperature–mortality associations under heterogeneous climatic conditions, rather than to define health-relevant thresholds or heat-health waves, which were addressed at the city scale. Using different modelling strategies reflects different analytical objectives and avoids artificial comparability between fundamentally distinct epidemiological quantities.

7 Since the late 1990s, it's been commonplace to say that there are more pets than babies in Barcelona. Indeed, in 2024, the city's pet census reached 85,565 pets, compared to 56,741 children aged between 0 and 4. The growth in the number of pets (unlike human births) has grown dramatically since the pandemic: more than 30%.

8 For the comparison between 1971 and 2025, an OLS model was performed with the estimate of the mean annual temperature (TM, or TX or TN) as the dependent variable and the year as the independent variable. The general criterion used in this paper is that when referring to the result of models (OLS for example), the warming is quantified with two decimal places.

9 For the period 1971-2024, the increase in the average temperature of Barcelona (3.29 °C) far exceeded the increase in the temperature of Spain, 1.96 °C (AEMET).

10 The estimate of the 2025-1971 increase differs slightly from the estimate made at the beginning of this section, due to the different methodology used: KTS regression instead of OLS.

11 It is worth remembering that the cubic models implemented for mortality and temperatures refer to the average of the entire period studied (2007-2018) of the smoothed data for each calendar day (15 days around the reference day (from lag−7 to lag +7).

12 Threshold_simple: first T where RR ≥ target. Threshold_sustained_0p5C / 1p0C: first T where RR ≥ target and remains so for at least 0.5 / 1.0 °C.

13 It should be noted, however, that the province of Barcelona is characterized by a high degree of temperature variation, a result of its geographical features (primarily distance from the sea and altitude). Therefore, the results should be considered with caution if they are to be extrapolated to the city of Barcelona. This issue is addressed in the discussion of the results section.

14 The proportion of older adults people in 2025 has been calculated in relation to the total population in 2011, in order to exclude population growth due to immigration. In fact, if we consider this indicator, Barcelona is the fourth of the 20 most populated cities with the most rapid aging process; however, the three preceding it (Bilbao, Valladolid and Gijón) have experienced a demographic decline between 2011 and 2025.

15 In Spain, the start of the baby boom was about 12 years later than in the US and Europe. The end of autarky (from 1957 onwards) marks the beginning of the Spanish baby boom, which lasted about 20 years.

16 The baby boomer generation likely has a very limited tendency to leave the city. The locational inertia of older generations, driven by the fact that a very high proportion of them own their homes (not rent), combined with their greater proximity to services (such as healthcare), suggests a strong tendency for these households to remain in the city center. Only the probably more significant tendency of immigrants (mostly renters) to leave the city in order to obtain more affordable housing could explain IDESCAT's conservative estimate.

17 The 2000-2025 model was created excluding 2003, a clear outlier.

18 The evolution of the average minimum temperatures between 1971 and 2025 (during the summer) indicated in the text refers to the results of the OLS model, not to the actual temperatures of those years.

19 For the last 25 years (2000-2025, excluding 2003), the increase in TN has been very pronounced: 2.21 °C, compared to 1.87 °C for TX.

20 The 2003 heatwave represents an exceptional large-scale event, both in magnitude and spatial extent, affecting most of Western Europe. Such synoptic-scale extremes disproportionately influence daytime heatwave metrics, which are more directly linked to regional atmospheric circulation. As a result, daytime heatwave trends exhibit greater sensitivity to individual extreme summers. In contrast, night-time heatwaves show a structurally consistent increase, reflecting the cumulative effect of background warming and urban heat retention processes.

21 For each isoclimatic zone, when the forecast maximum temperature is higher than the threshold temperature for three consecutive days, the difference is added together. A low risk is determined if the sum is less than 3.5 degrees Celsius, a medium risk if the sum is between 3.5 and 7 degrees Celsius, and a high risk if it exceeds 7 degrees Celsius. The municipality of Barcelona is located in the ”Barcelona Littoral" zone.

22 Although the ISCIII definition yields a slightly higher attributable fraction during exposed days, this does not translate into a higher population impact. In contrast, nighttime heathealth waves show substantially larger attributable mortality due to their much higher frequency and duration.

23 Currently, this percentile corresponds to 34.5°C and 27.4°C. The latter is significantly higher than that established by the SMC (26.5°C), which demonstrates the considerable instability of the 98th percentile in the context of current warming.
